# Prenatal Opioid Exposure Impairs Endocannabinoid and Glutamate Transmission in the Dorsal Striatum

**DOI:** 10.1523/ENEURO.0119-22.2022

**Published:** 2022-04-19

**Authors:** Gregory G. Grecco, Braulio Muñoz, Gonzalo Viana Di Prisco, Emma H. Doud, Brandon M. Fritz, Danielle Maulucci, Yong Gao, Amber L. Mosley, Anthony J. Baucum, Brady K. Atwood

**Affiliations:** 1 Department of Pharmacology and Toxicology, Indiana University School of Medicine, Indianapolis, Indiana 46202; 2 Medical Scientist Training Program, Indiana University School of Medicine, Indianapolis, Indiana 46202; 3 Department of Biochemistry and Molecular Biology, Indiana University School of Medicine, Indianapolis, Indiana 46202; 4 Center for Proteome Analysis, Indiana University School of Medicine, Indianapolis, Indiana 46202; 5 Center for Computational Biology and Bioinformatics, Indiana University School of Medicine, Indianapolis, Indiana 46202; 6 Department of Biology, Indiana University-Purdue University, Indianapolis, Indiana 46202; 7 Stark Neurosciences Research Institute, Indiana University School of Medicine, Indianapolis, Indiana 46202

**Keywords:** endocannabinoid, methadone, plasticity, prenatal opioid exposure, proteomics

## Abstract

The opioid crisis has contributed to a growing population of children exposed to opioids during fetal development; however, many of the long-term effects of opioid exposure on development are unknown. We previously demonstrated that opioids have deleterious effects on endocannabinoid plasticity at glutamate synapses in the dorsal striatum of adolescent rodents, but it is unclear whether prenatal opioid exposure produces similar neuroadaptations. Using a mouse model of prenatal methadone exposure (PME), we performed proteomics, phosphoproteomics, and patch-clamp electrophysiology in the dorsolateral striatum (DLS) and dorsomedial striatum (DMS) to examine synaptic functioning in adolescent PME offspring. PME impacted the proteome and phosphoproteome in a region- and sex-dependent manner. Many proteins and phosphorylated proteins associated with glutamate transmission were differentially abundant in PME offspring, which was associated with reduced glutamate release in the DLS and altered the rise time of excitatory events in the DMS. Similarly, the intrinsic excitability properties of DMS neurons were significantly affected by PME. Last, pathway analyses revealed an enrichment in retrograde endocannabinoid signaling in the DLS, but not in the DMS, of males. Electrophysiology studies confirmed that endocannabinoid-mediated synaptic depression was impaired in the DLS, but not DMS, of PME-males. These results indicate that PME induces persistent neuroadaptations in the dorsal striatum and could contribute to the aberrant behavioral development described in offspring with prenatal opioid exposure.

## Significance Statement

Rewarding drugs, including opioids, are known to disrupt endocannabinoid and glutamate signaling in the dorsal striatum of mature rodents, which may underlie maladaptive behavioral processes associated with addiction. Given the growing population of children with prenatal opioid exposure, we sought to determine whether prenatal opioid exposure produces similar neuroadaptations in the dorsal striata of offspring. Using a mouse model of prenatal methadone exposure (PME), we discovered endocannabinoid-mediated synaptic depression was impaired in the dorsolateral striatum and glutamate signaling was disrupted in the dorsolateral and dorsomedial striata of adolescent PME offspring. These neuroadaptations likely alter the functional output of the dorsal striatum, which may contribute to the aberrant behavioral development and altered reward phenotype often associated with prenatal opioid exposure.

## Introduction

While the opioid crisis has afflicted numerous populations across various demographics (Mattson et al., 2021), the pregnant woman and her developing infant represent a uniquely vulnerable population. Maternal diagnosis of opioid use disorder (OUD) at delivery rose 131% from 2010 to 2017 in the United States, contributing to an 82% rise in the diagnosis of neonatal abstinence syndrome (also referred to as neonatal opioid withdrawal syndrome; Hirai et al., 2021). To address the growing concerns regarding the impact of prenatal opioid exposure on neurodevelopment, our laboratory developed a clinically relevant model of prenatal methadone exposure (PME) to investigate various offspring outcomes (Grecco et al., 2021a). Offspring born of this model demonstrate high levels of methadone within the brain, opioid withdrawal signs at birth, and disruptions in behavioral development without any confounding changes in maternal/pregnancy characteristics or maternal care behavior (Grecco et al., 2021a).

Various animal models have suggested that prenatal opioid exposure augments the sensitivity to drug reward later in life including an increase in opioid consumption, self-administration, and conditioned place preference (Grecco and Atwood, 2020). Our PME model demonstrates several sex-dependent alterations in alcohol reward. PME-males exhibited increased alcohol consumption and aversion-resistant alcohol consumption, while we observed enhanced alcohol-induced hyperactivity in PME-females (Grecco et al., 2022). These findings might suggest that PME produces persistent neuroadaptations in key brain regions associated with reward behavior, such as the striatum, which may predispose offspring to show greater drug seeking and drug taking-related behaviors when re-exposed to drugs later in life. Indeed, previous work has revealed that prenatal opioid exposure may disrupt dopaminergic (De Vries et al., 1991), opioidergic, (Tempel and Espinoza, 1992; Tempel et al., 1995; Chiou et al., 2003), and cholinergic (Guo et al., 1990; Robinson et al., 1991) signaling in the striatum of neonatal rodents. Unfortunately, previous studies seldom examine animals beyond the preweaning period, a time period when opioids may still be present in the offspring and continue to exert pharmacological effects. Previous work has not assessed dorsal striatal subregions, which represents a critical knowledge gap given that subregions of the striatum play diverse roles in addiction processes. While the dorsal striatum in general is known to mediate drug seeking and consumption, the dorsolateral striatum (DLS) plays a more prominent role in habitual, compulsive, and aversion-resistant drug seeking and consumption, while dorsomedial striatum (DMS) regulates goal-oriented drug seeking and consumption (Corbit et al., 2012; Fanelli et al., 2013; Renteria et al., 2018; Giuliano et al., 2019; Roltsch Hellard et al., 2019; Cheng et al., 2021). The DLS and DMS are targets for the deleterious effects of both alcohol and opioids when administered to adolescent and adult rodents (DePoy et al., 2013; Atwood et al., 2014; Corbit et al., 2014; Muñoz et al., 2018, 2020; Blackwood et al., 2019). We have shown that oxycodone disrupts endocannabinoid-mediated long-term synaptic depression (eCB-LTD) of glutamate transmission in the dorsal striatum when administered to adolescent animals (Atwood et al., 2014). This eCB-LTD underlies habitual learning processes that are accelerated by drugs of abuse (Nazzaro et al., 2012; DePoy et al., 2013; Gremel et al., 2016; Renteria et al., 2018). It remains unclear whether passive prenatal exposure to opioids produces similar neuroadaptations in dorsal striatal circuitry. This is important as prenatal opioid exposure-induced neuroadaptations in dorsal striatal circuity may underlie increased drug and alcohol behavioral responses in later life.

To elucidate potential neural mechanisms contributing to the alterations in reward behavior in our model of PME, a quantitative proteomics and phosphoproteomic analysis was completed in the DLS and DMS. This multiomic assessment spurred further investigation of neurotransmission and plasticity in medium spiny neurons (MSNs), the predominant cell type of the dorsal striatum, using brain slice whole-cell patch-clamp electrophysiology in adolescent offspring with PME. Our findings revealed that PME induces widespread changes to the dorsal striatal proteomic and phosphoproteomic landscapes and disrupts neurotransmission, including disruptions in eCB-LTD in DLS, which persist into adolescence.

## Materials and Methods

### Animals and model generation

For animal studies, all guidelines established by the National Institutes of Health were used, and the Indiana University School of Medicine Institutional Animal Care and Use Committee approved all research and protocols. To generate PME and prenatal saline-exposed (PSE) control offspring, 8-week-old female C57BL/6J mice were randomly assigned to receive treatment with either saline (10 ml/kg) or oxycodone (10 mg/kg, s.c., twice on day 1; 20 mg/kg, s.c., twice on day 2; and 30 mg/kg, s.c., twice a day on days 3–9) for 9 d pregestationally to induce opioid dependency before initiating “treatment” for OUD. All saline or oxycodone doses were administered subcutaneously twice daily at least 7 h apart. Oxycodone-dependent mice were then transitioned to methadone (10 mg/kg, s.c., twice a day), while saline-treated animals continued to receive saline injections. Five days after methadone treatments began,& following the initiation of methadone treatment, a C57BL/6J male mouse was placed into the cage of each female for 4 d. Methadone or saline treatments continued throughout the remainder of pregnancy and postnatal period up to weaning. This model was designed to replicate a typical pattern of opioid use in a pregnant woman who is first dependent on oxycodone and then initiates methadone pharmacotherapy for their OUD and becomes pregnant while taking methadone (Tolia et al., 2015; Duffy et al., 2018). We have previously characterized methadone levels in dams and offspring of this model (Grecco et al., 2021a), and this dose in mice is purported to be within the human therapeutic range (Devidze et al., 2008). Although offspring born of this model display withdrawal at birth and altered sensorimotor development, we have not observed significant effects of opioid treatment on maternal care, pregnancy characteristics, or litter characteristics (Grecco et al., 2021a). For further description of the model generation, please see the study by Grecco et al. (2021a). For the following studies, male and female mice (between 5 and 7 weeks old) were used with no more than two per sex from any given litter to minimize litter effects. Given that mice are weaned at approximately postnatal day 28 (P28), offspring have not any opioid exposure via breast milk for at least 7 d. Experimenters were blinded to exposure group for data collection of all studies.

### Proteomics and phosphoproteomics

#### Protein preparation

Sample preparation, mass spectrometry (MS) analysis, bioinformatics, and data evaluation for quantitative proteomics and phosphoproteomics experiments were performed in collaboration with the Indiana University School of Medicine Center for Proteome Analysis, similar to those previously completed (Grecco et al., 2021b).

Animals were rapidly decapitated without anesthesia between 1:00 and 4:00 P.M. during the light cycle. Blinded researchers collected the dorsal striatum bilaterally and bisected it to approximately separate DLS from DMS. Tissue was immediately snap frozen in isopentane on dry ice and stored until later processing. Flash-frozen brain lysates were homogenized in 1 ml of 9 m urea [catalog #16199, Chemical Entities of Biological Interest (CHEBI)] in 100 mm Tris, pH 8.0 (catalog #9754, CHEBI), 1× using a BeadBug 6 (catalog #D1036, Benchmark Scientific; 3 mm zirconium beads, catalog #D1032-30, Benchmark Scientific; 10 rounds of 30 × 30 s, at 4°C). Samples were next sonicated in 1.5 ml Micro Tubes (TPX Plastic for Sonication, Diagenode) using a Bioruptor sonication system (North America catalog #B01020001, Diagenode USA) with 30 s on/off cycles for 15 min in a water bath at 4°C. After subsequent centrifugation at 12,000 relative centrifugal force for 20 min, protein concentrations were determined by Bradford protein assay (catalog #5000006, BIO-RAD). A 100 μg equivalent of protein from each sample were then treated with 5 mm Tris(2-carboxyethyl)phosphine hydrochloride (catalog #C4706, Sigma-Aldrich) to reduce disulfide bonds, and the resulting free cysteine thiols were alkylated with 10 mm chloroacetamide (catalog #C0267, Sigma-Aldrich). Samples were next diluted with 100 mm Tris HCl (Sigma-Aldrich Cat No: 10812846001) to a final urea concentration of 2 m to carry out trypsin/Lys-C-based overnight protein digestion to derive peptides (1:100 protease/substrate ratio, mass spectrometry grade; catalog #V5072, Promega; Levasseur et al., 2019; Li et al., 2020).

#### Peptide cleanup and tandem mass tag isobaric labeling

Digestions were quenched with 0.4% trifluoroacetic acid (TFA; v/v; catalog #91699, Fluka), and the resultant peptides were desalted by solid-phase extraction using Sep-Pak Vac cartridges C18 cartridges (catalog #WAT054955, Waters), lyophilized O/N, and resuspended in 55 μl of 50 mm triethylammonium bicarbonate (catalog #T7408, Sigma-Aldrich), pH 8.5. Peptides were quantified using Quantitative Colorimetric Peptide Assay (catalog #23275, Thermo Fisher Scientific) to ensure equivalent concentrations across each set of samples before being covalently labeled with TMTpro Isobaric Label Reagent 16-plex (catalog #44520, lot VI310352, Thermo Fisher Scientific) at a 1:7 peptide to TMTpro ratio. After 1 h of incubation, the labeling reaction was quenched with 0.3% hydroxylamine (v/v) for 15 min before combining the samples. The multiplexed sample was concentrated to dryness in vacuum centrifuge, reconstituted with 0.1% TFA aqueous (v/v), desalted via Waters Sep-Pak Vac cartridges as before, and lyophilized.

#### Peptide purification and labeling

Phosphopeptides were enriched using a High-Select TiO_2_ Phosphopeptide Enrichment Kit (catalog #A32993, Thermo Fisher Scientific). After preparing spin tips, labeled and mixed peptides were repeatedly applied to TiO_2_ spin tips and eluted as per manufacturer instructions. The flow-through from each tip was saved for global proteomics.

#### High pH basic fractionation

The flow-through from phosphoproteomics enrichment was lyophilized, resuspended in 150 μl of 10 mm formate, pH 10, and fractionated using an offline UltiMate 3000 HPLC system (Thermo Fisher Scientific) with an XBridge C18 column (3.5 μm × 4.6 mm × 250 mm; catalog #186003943, Waters; buffer A: 10 mm formate, pH 10; buffer B: 10 mm formate, pH 10; 95% acetonitrile, gradient 1 ml/min 0–15% buffer B over 5 min, 15–20% buffer B over 5 min, 20–35% buffer B over 75 min, 35–50% buffer B over 5 min, 50–60% buffer B over 10 min, and a 6 min hold at 60% buffer B). Fractions were collected continuously every 60 s into 96-well plates. Initial and late fractions with minimal material were combined and lyophilized. The remaining fractions were concatenated into 24 fractions, dried down, and resuspended in 50 μl of 0.1% formic acid (FA; catalog #30751, CHEBI) before online liquid chromatography (LC)-MS (Batth et al., 2014; Bai et al., 2017).

#### Nano-LC-tandem MS analysis

Nano-LC-tandem MS (MS/MS) analyses were performed on an EASY-nLC HPLC system (catalog #014993, Thermo Fisher Scientific) coupled to an Orbitrap Fusion Lumos Mass Spectrometer (Thermo Fisher Scientific). One-third of each fraction was loaded onto a reversed phase EASY-Spray C18 column (2 μm, 100 Å, 75 μm × 50 cm; catalog #ES802A, Thermo Fisher Scientific) at 400 nl/min. One-fifth of the phosphopeptides and one-tenth of each global peptide fraction were analyzed per run. Peptides were eluted from 4% to 28% with mobile phase B [mobile phase A: 0.1% FA, water; mobile phase B: 0.1% FA, 80% acetonitrile (catalog #LS122500, Thermo Fisher Scientific) over 160 min; 28−35% mobile phase B over 5 min; 35−50% mobile phase B for 14 min; and dropping from 50% to 10% mobile phase B over the final 1 min]. The mass spectrometer method was operated in positive ion mode with a 4 s cycle time data-dependent acquisition method with advanced peak determination and Easy-IC (internal calibrant). Precursor scans [mass/charge ratio (m/z), 400–1750] were performed with an Orbitrap resolution of 120,000, RF lens 30%, maximum inject time of 50 ms, and standard automatic gain control (AGC) target, including charges of 2–6 for fragmentation with 60 s dynamic exclusion. MS/MS scans were performed with a fixed first mass of 100 m/z, 34% fixed collision energy (CE), 50,000 resolution, 20% normalized AGC target, and dynamic maximum injection time (IT). The data were recorded using Xcalibur (version 4.3) software (Thermo Fisher Scientific).

#### Proteome and phosphoproteome analysis

The resulting RAW files were analyzed in Proteome Discover 2.4 (Thermo Fisher Scientific; RRID:SCR_014477) with a *Mus musculus* UniProt FASTA plus common contaminants (reviewed and unreviewed sequences were downloaded October 20, 2019). Quantification methods used isotopic impurity levels available from Thermo Fisher Scientific. SEQUEST HT searches were conducted with a maximum number of three missed cleavages; precursor mass tolerance of 10 ppm; and a fragment mass tolerance of 0.02 Da. Static modifications used for the search were as follows: (1) carbamidomethylation on cysteine residues; and (2) TMTpro label on lysine residues and the N termini of peptides. The dynamic modifications used for the search were oxidation of methionines and acetylation of N termini. The percolator false discovery rate (FDR) was set to a strict setting of 0.01 and a relaxed setting of 0.05. IMP-ptm-RS node was used for all modification site localization scores. Values from both unique and razor peptides were used for quantification. In the consensus workflows, peptides were normalized by total peptide amount with no scaling. Data shown is for PME/PSE abundance value ratios (ARs). Resulting grouped abundance values for each sample type, AR values, and respective *p*-values (*t* test) from Proteome Discover were exported to Microsoft Excel. The full raw datasets can be found at https://github.com/gggrecco/Dorsal-Striatum-Omics, in addition to the extended data files.

#### Network and enrichment analyses

All analyses are presented as PME relative to PSE (e.g., log2 abundance ratios of PME/PSE). To determine hub proteins and phosphorylated proteins, differentially expressed proteins were first sorted and filtered for *p* values < 0.05, and hub protein analysis of this differentially expressed protein and phosphorylated network was completed using CytoScape with the cytoHubba plugin (Chin et al., 2014). Hubs were sorted by the maximal clique centrality (MCC), which is a network scoring method that ranks essential nodes in a network and has been demonstrated to be more robust than other measures (Chin et al., 2014). For pathway analysis of global proteome data, the UniProt Accessions of all differentially expressed proteins (*p* < 0.05) were submitted to the g:Profiler g:GOst Functional Profiling platform (Raudvere et al., 2019). For settings, all known genes were selected for the statistical domain scope and the significance threshold was set to a strict Benjamini–Hochberg FDR of <0.01. The term size was filtered to between 5 and 2000. The full results of the pathway analysis are provided in the extended data files. A kinase-substrate enrichment analysis (KSEA) of the phosphoproteomics data were performed using the KSEA application (https://casecpb.shinyapps.io/ksea/; Wiredja et al., 2017). All identified phosphopeptides with quantified abundance ratios (PME/PSE) and confirmed phosphosite modifications were used for the KSEA. PhosphoSitePlus + NetworKIN (NetworKIN score cutoff, 2) were used as the kinase-substrate dataset. Results were FDR corrected (<0.05), and a *z* score of enrichment was calculated to determine the normalized magnitude of upregulation or downregulation (PME vs PSE). The full results of the KSEA are provided in the supplementary information. The kinase scores resulting from the KSEA analysis were exported to Coral and overlayed onto kinome trees to better visualize patterns in kinase regulation across brain region and sex where branches were set to represent the significance level, node color represents the *z* score of enrichment, and node size represents the size of enrichment (absolute value of *z* score; Metz et al., 2018). Venn diagram plots for the overlap in proteins and phosphopeptides were generated with Venny 2.1 (https://bioinfogp.cnb.csic.es/tools/venny/index.html). The full hub protein and enrichment analyses can be found at https://github.com/gggrecco/Dorsal-Striatum-Omics in addition to the extended data files.

### Electrophysiology

#### Slice preparation and recording conditions

Mice were anesthetized with isoflurane and rapidly decapitated to excise brain tissue. Brains were quickly transferred to an ice-cold, oxygenated (95% CO_2_/5% O_2_ bubbled) cutting solution containing (mm): 30 NaCl, 4.5 KCl, 1 MgCl_2_, 26 NaHCO_3_, 1.2 NaH_2_PO_4_, 10 glucose, and 194 sucrose. Coronal brain slices containing the striatum were taken at 280 μm using a VT1200S vibratome (Leica). These sections were placed into a holding chamber held at 32°C filled with oxygenated artificial CSF (aCSF) containing (mm): 124 NaCl, 4.5 KCl, 2 CaCl2, 1 MgCl2, 26 NaHCO3, 1.2 NaH_2_PO_4_, and 10 glucose. Slices were incubated at 32°C for 1 h before being transferred to room temperature until the time of recording.

Whole-cell voltage and current-clamp recordings were acquired using a Multiclamp 700B amplifier and Digidata 1550B (Molecular Devices). Brain slices, which were held at 32°C and continuously perfused with oxygenated aCSF at a rate of ∼1.5 ml/min, were moved to a recording chamber for recordings. Slices were visualized on a BX51WI microscope (Olympus). MSNs in the DMS and DLS were confirmed by their membrane resistance (80–400 MΩ) and capacitance (100–200 pF), as previously reported (Gertler et al., 2008). Borosilicate glass recording pipettes of 2–4 MΩ were filled with the appropriate internal solutions (see below; adjusted to 295–310 mOsm). All recordings were filtered at 2.2 kHz and digitized at 10 kHz. MSNs were in voltage-clamp mode and held at −60 mV. Data were acquired using Clampex 10 software (Molecular Devices). Series resistance was continuously monitored, and only cells with a stable access resistance (<25 MΩ, and that did not change ≥15%) were included for data analysis.

For recordings of glutamate transmission, excitatory currents were isolated by adding 50 μm picrotoxin to the aCSF.

#### Excitatory transmission

The internal solution for excitatory recordings contained the following (mm): 120 CsMeSO3, 5 NaCl, 10 TEA-Cl, 10 HEPES, 5 lidocaine bromide, 1.1 EGTA, 0.3 Na-GTP, and 4 Mg-ATP. After a stabilization period of 7–10 min, spontaneous EPSCs (sEPSCs) were measured over the course of 3 min gap-free recordings. Parameters measured included the following: sEPSC amplitude, frequency, rise time, and decay constant.

The ratio of the glutamate-driven AMPA receptor to NMDA receptor current was assessed in voltage-clamp mode. A Teflon-coated bipolar stimulating electrode (Plastics One) was placed at the border of the white matter of the external capsule for recordings in the DLS. For DMS recordings, the stimulating electrode was placed at the border of the overlying corpus callosum and DMS, and recordings were performed from MSNs just lateral to the dorsolateral-most point of the lateral ventricle. EPSCs were evoked via a DS3 Isolated Current Stimulator (Digitimer USA). The intensity of stimulation was adjusted to produce evoked EPSCs of 200–400 pA in amplitude. To measure AMPA/NMDA receptor-mediated current ratios, the cell was first held at −80 mV and AMPAR-mediated EPSCs were electrically evoked. For the NMDAR current, the cell was then held at +40 mV and an EPSC was again evoked. As the AMPAR component of EPSCs at −80 mV was not apparent 100 ms following the electrical stimulus (i.e., the measured current returned to baseline), the NMDAR-mediated portion of the EPSC at +40 mV was calculated as the average of the measured current over the following 25 ms (100–125 ms poststimulus), as we have previously done (Fritz et al., 2018). sEPSCs were measured first, so that repeated stimulation required by evoked response recordings did not influence sEPSC parameters.

#### Excitability

For excitability recordings, a K-gluconate internal solution was used containing the following (mm): 4 KCl, 10 HEPES, 4 MgATP, 0.3 NaGTP, 10 phosphocreatine, and 126 K-gluconate. Cells were recorded in current-clamp mode and allowed to sit at their natural resting membrane potential (RMP). Increasing current steps (−200 to +400 pA in 50 pA increments) were injected for 500 ms every 10 s. The following excitability parameters were measured: RMP, input resistance, voltage sag, threshold potential, action potential (AP) peak, AP half-width, and AP frequency. The data from the first current step that produced APs were used to calculate threshold potential, AP peak, and AP half-width parameters.

#### Endocannabinoid-mediated long-term depression

Stimulating electrodes were placed in the same positions as were used for the measures of AMPA and NDMA receptor-mediated currents. EPSCs were evoked every 20 s, and the intensity was adjusted until a stable response between −200 and −400 pA was observed. eCB-LTD was induced by depolarizing the postsynaptic MSN to 0 mV in combination with high-frequency stimulation (HFS; four trains of 1 s, 100 Hz stimulation separated by 10 s) or with bath application of 1 μm WIN55,212–2 for 10 min.

#### Electrophysiology data processing and statistical analysis

All excitability, AMPA/NMDA ratio, and LTD data were processed via pClamp 10.6 software (Molecular Devices). sEPSC data were processed via MiniAnalysis software (Synaptosoft). Statistical analyses were completed in Prism 9 (GraphPad). DMS and DLS data were analyzed separately. All sample sizes indicated in figures for electrophysiological experiments represent biological replicates. For LTD recordings, one neuron was recorded per brain slice, and all experiments involved recordings are from at least three mice per sex per exposure. Student’s *t* tests were implemented when only two groups were being assessed (LTD data) with paired *t* tests being used for repeated measures (LTD data). When multiple groups were being compared, ANOVAs were used that included exposure (PME/PSE), sex (male/female), and repeated measures when applicable (e.g., frequency of action potentials at various current steps). The significance level for all analyses was set at *p *<* *0.05 and Sidak’s *post hoc* test statistics were run where applicable.

## Results

### Differential protein and phosphopeptide abundance and network enrichment

To initiate our exploration into possible molecular pathways uniquely disrupted in the dorsal striatum of PME offspring, we collected DLS and DMS tissue from adolescent male and female PME and PSE offspring for quantitative proteomic and phosphoproteomic analyses.

**Figure 1.**
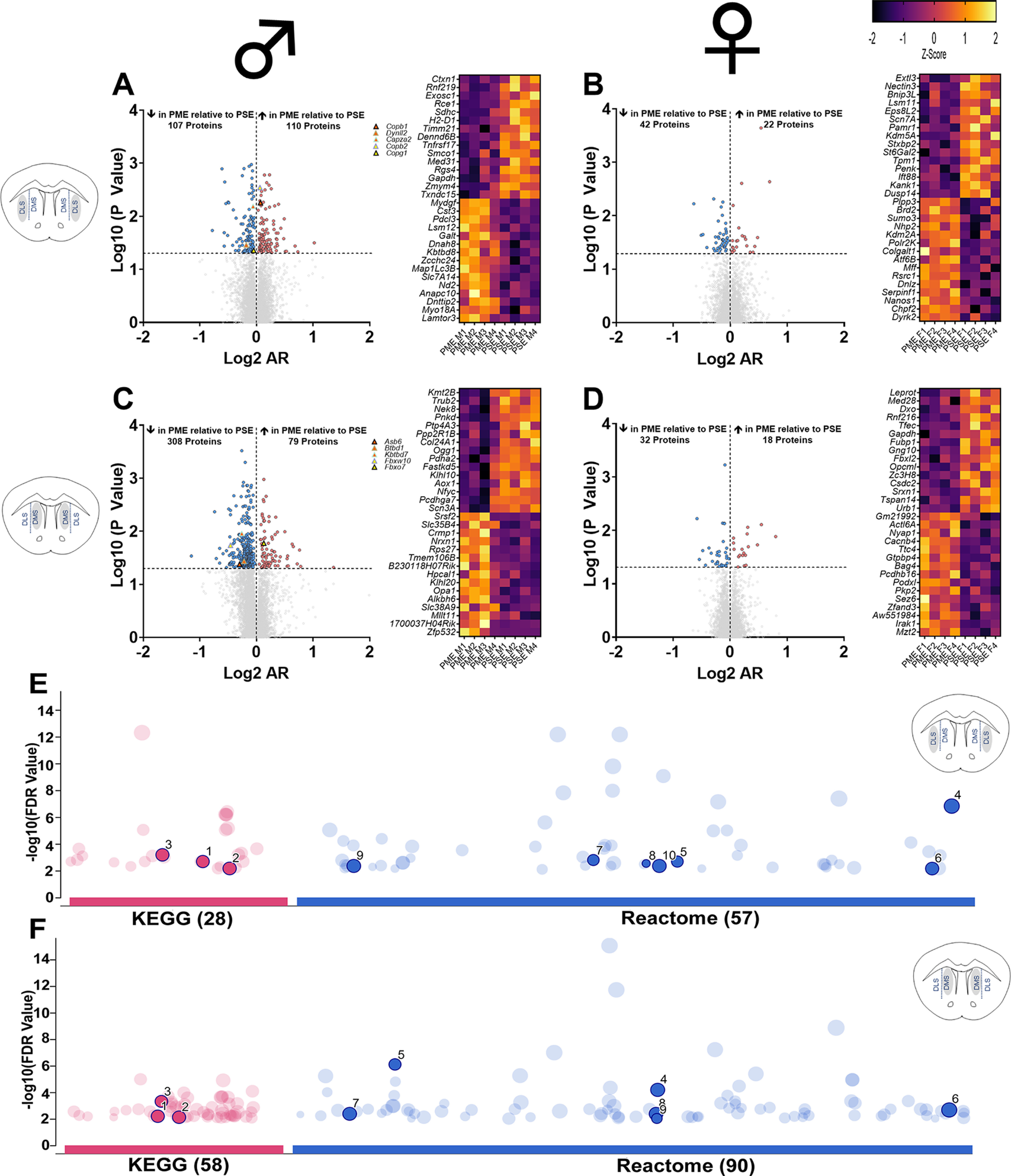
PME changes the dorsal striatal proteome and leads to numerous network changes ***A–D***, Volcano plots (left; blue circles, proteins decreased in PME vs PSE; red circles, proteins increased in PME vs PSE, which reach the level of significance) and heatmaps (right) identifying the top 30 differentially expressed proteins in the DLS of males (***A***) and females (***B***), and in the DMS of males (***C***) and females (***D***; Extended Data Figs. 1-1, 1-2, full dataset in the DLS and DMS, respectively). The top five hub proteins identified from the network of differentially expressed proteins are plotted as triangles on volcano plots. For more information, see Table 1 and Extended Data Figure 1-3. ***E***, ***F***, Pathway analysis of enriched KEGG and Reactome terms (represented as pink and blue dots, respectively) among the significant differentially expressed proteins for the DLS in males (***E***) and DMS in males (***F***). Highlighted and numbered terms include retrograde endocannabinoid signaling (1), alcoholism (2), cGMP–PKG signaling pathway (3), vesicle-mediated transport (4), opioid signaling (5), transmission across chemical synapses (6), L1CAM interactions (7), NGF-stimulated transcription (8), axon guidance (9), and nervous system development (10) in the DLS (***E***); and cGMP–PKG signaling pathway (1), axon guidance (2), sphingolipid signaling pathway (3), neuronal system (4), cell–cell communication (5), vesicle-mediated transport (6), axon guidance (7), nervous system development (8), and neurexins and neuroligins (9) the DMS (***F***; for full enrichment analysis results, see Extended Data Fig. 1-4; *n* = 8 PME (4 males, 4 females) and 8 PSE (4 males, 4 females).

Overall, we identified many more proteins that were significantly differentially expressed between prenatal exposure groups (PME vs PSE) in males compared with females. In the DLS, 8440 proteins were quantified with 217 and 64 differentially expressed (*p* < 0.05) in males and females, respectively (Fig. 1*A*,*B*, left). In the DMS, 9196 proteins were identified with 387 and 50 differentially expressed (*p* < 0.05) in males and females, respectively (Fig. 1*C*,*D*, left). Heatmaps of the top 30 differentially expressed proteins were generated to visualize the diversity of proteins identified (Fig. 1*A–D*, right). Hub proteins within the network of differentially expressed proteins (presented as triangles on their respective volcano plots) were discovered using the Cytoscape plugin cytoHubba and sorted by MCC, which identifies key proteins of the network with both high and low levels of degree (Chin et al., 2014). The identities of all hub proteins for each respective network of differentially expressed proteins are presented in Table 1. In the DLS of males, the vesicle coating proteins (Copb1, Copb2, Copg1) and cytoskeletal structural proteins (Capza2, Dynll2) demonstrated the greatest MCC (Fig. 1*A*, left, Table 1). For the network of differentially expressed proteins in the DMS of males, proteins involved in the ubiquitination and proteasomal degradation of target proteins were identified as top hub proteins (Btbd1, Asb6, Kbtbd7, Fbxw10, Fbxo7; Fig. 1*A*, left, Table 1). However, the differential protein network in the DLS and DMS of females did not exhibit enough edges to calculate hub proteins indicating limited protein network enrichment.

**Table 1:** Hub proteins from differential protein and phosphopeptide expression networks in the DMS and DLS of males and females

Gene	Protein	MCC
DLS hub proteins for global proteome network in males		
*Copb1*	Coatomer subunit β-1	242
*Dynll2*	Dynein light chain 2, cytoplasmic	241
*Capza2*	F-actin-capping protein subunit α-2	240
*Copb2*	Coatomer subunit β-2	240
*Copg1*	Coatomer subunit γ-1	240
DMS hub proteins for global proteome network in males		
*Btbd1*	BTB/POZ domain-containing protein 1	5760
*Asb6*	Ankyrin repeat and SOCS box protein 6	5760
*Kbtbd7*	Kelch repeat and BTB (POZ) domain containing 7	5760
*Fbxw10*	F-box/WD repeat-containing protein 10	5760
*Fbxo7*	F-box only protein 7	5760
DLS hub proteins for phosphoproteome network in males		
*Sptbn1*	Spectrin beta chain, nonerythrocytic 1	7
*Ank2*	Ankyrin-2	6
*Ank3*	Ankyrin-3	6
*Ank1*	Ankyrin-1	6
*Rpl37*	60S ribosomal protein L37	6
DLS hub proteins for phosphoproteome network in females		
*Gapvd1*	GTPase-activating protein and VPS9 domain-containing protein 1	6
*Reps1*	RalBP1-associated Eps domain-containing protein 1	6
*Arrb1*	β-Arrestin-1	6
*Dnm3*	Dynamin-3	6
*Dlg2*	Disks large homolog 2	1
DMS hub proteins for phosphoproteome network in males		
*Hspa8*	Heat shock cognate 71 kDa protein	4
*Cask*	Peripheral plasma membrane protein CASK	3
*Epb4.1l1*	Band 4.1-like protein 1	3
*Epn2*	Epsin-2	2
*Camk2b*	Calcium/calmodulin-dependent protein kinase type II subunit β	2
DMS hub proteins for phosphoproteome network in females		
*Vcl*	Vinculin	4
*Cyfip1*	Cytoplasmic FMR1-interacting protein 1	2
*Lrrc7*	Leucine-rich repeat-containing protein 7	2
*Arhgef2*	Rho guanine nucleotide exchange factor 2	1
*Cask*	Peripheral plasma membrane protein CASK	1

10.1523/ENEURO.0119-22.2022.f1-1 Figure 1-1 Global proteome DLS. This file contains the full proteomics results for males and females in the DLS. Download Figure 1-1, XLS file.

**Figure 2.**
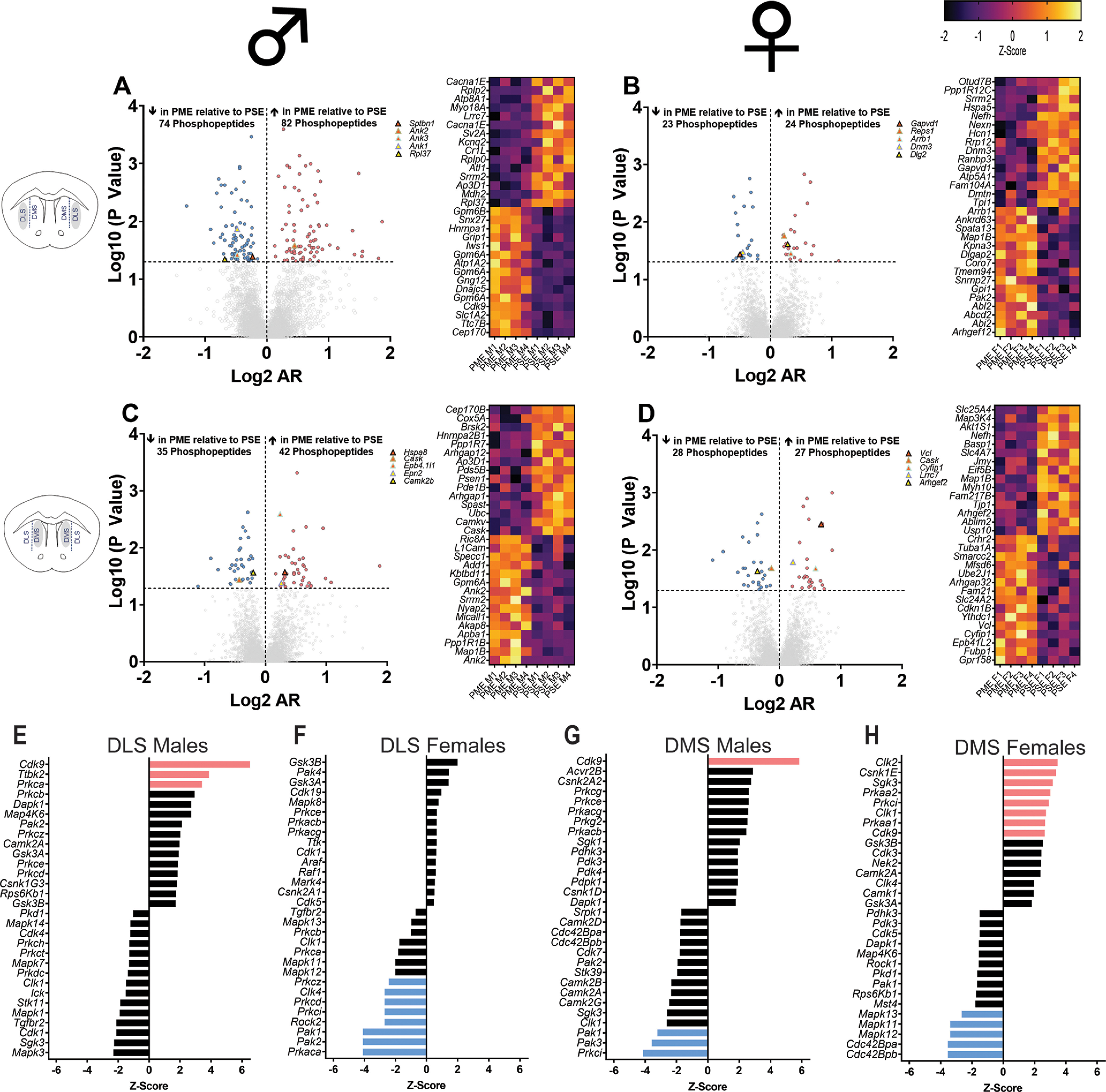
PME alters the dorsal striatal phosphoproteome and kinase pathways. ***A–D***, Volcano plots (left; blue circles, phosphopeptides decreased in PME vs PSE; red circles, phosphopeptides increased in PME vs PSE, which reach the level of significance) and heatmaps (right) identifying the top 30 differentially expressed phosphopeptides the DLS in males (***A***) and females (***B***) and DMS males (***C***) and females (***D***; Extended Data Figs. 2-1, 2-2, full dataset in the DLS and DMS, respectively) Phosphorylated protein hubs of the network of differentially expressed phosphorylated proteins are identified as various triangle symbols on each respective volcano plot (Table 1, Extended Data Fig. 1-3 for more information). ***E–H***, The results of a kinase substrate enrichment analysis demonstrating the top 30 kinases dysregulated. ***E–H***, Right, Blue bars represent kinases decreased in PME versus PSE and red bars represents proteins increased in PME versus PSE, which reach the level of significance for DLS in males (***E***) and females (***F***) and DMS males (***G***) and females (***H***). Extended Data Figure 2-3, KSEA scores; Extended Data Figures 2-4, 2-5, 2-6, 2-7, coral treeplots. *n* = 8 PME mice (4 males, 4 females) and 8 PSE mice (4 males, 4 females).

10.1523/ENEURO.0119-22.2022.f1-2 Figure 1-2 Global proteome DMS. This file contains the full proteomics results for males and females in the DMS. Download Figure 1-2, XLS file.

**Figure 3.**
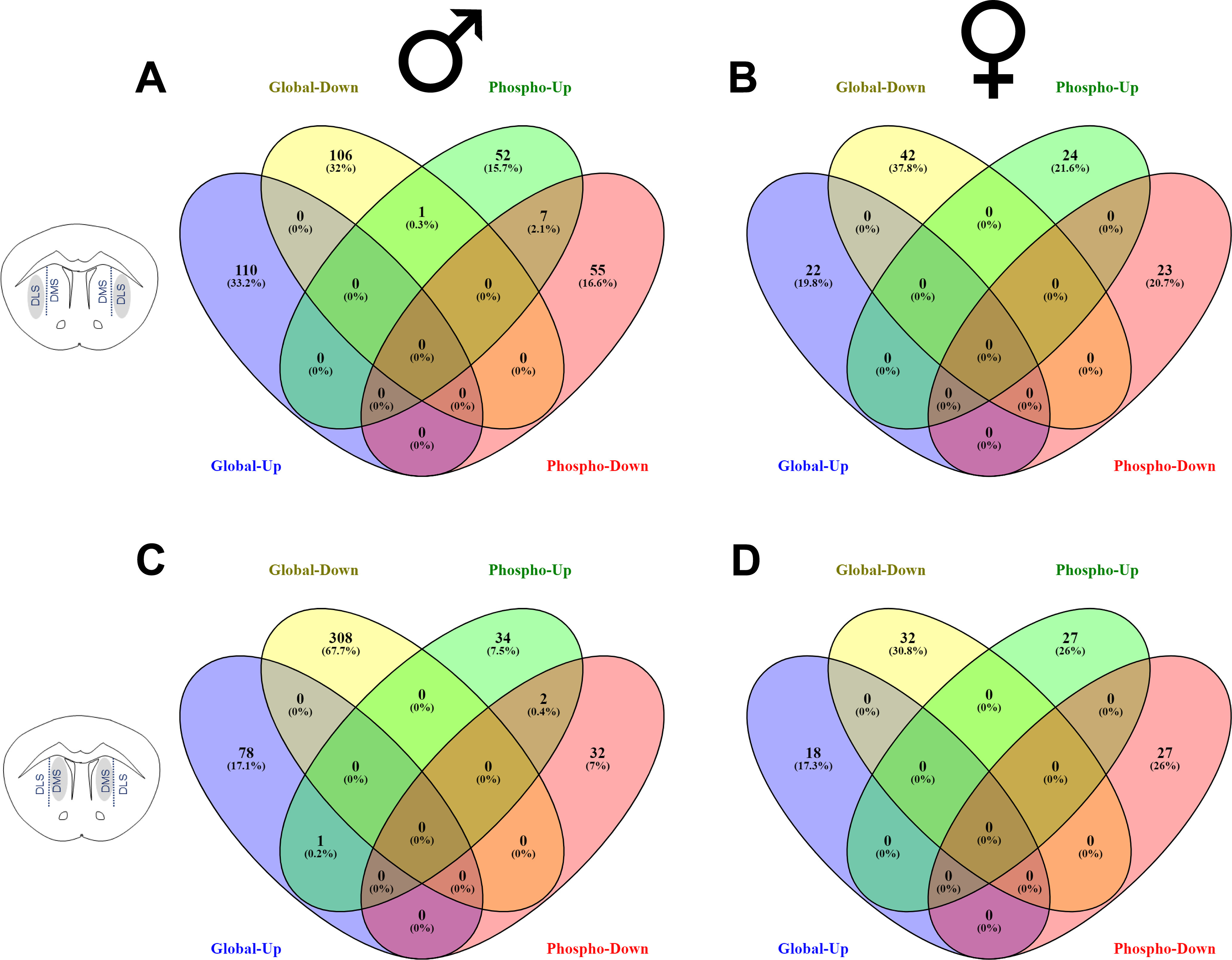
Overlap in differentially abundant proteins and phosphopeptides. ***A–D***, Venn diagrams demonstrating the overlap in significantly increased global proteins (blue), decreased global proteins (yellow), increased phosphopeptides (green), and decreased phosphopeptides (red) in PME relative to PSE mice for the DLS of males (***A***) and females (***B***) and DMS of males (***C***) and females (***D***).

10.1523/ENEURO.0119-22.2022.f1-3 Figure 1-3 Hub protein analysis. This file contains the full hub protein analysis results for males and females in both the DLS and DMS. Download Figure 1-3, XLS file.

**Figure 4.**
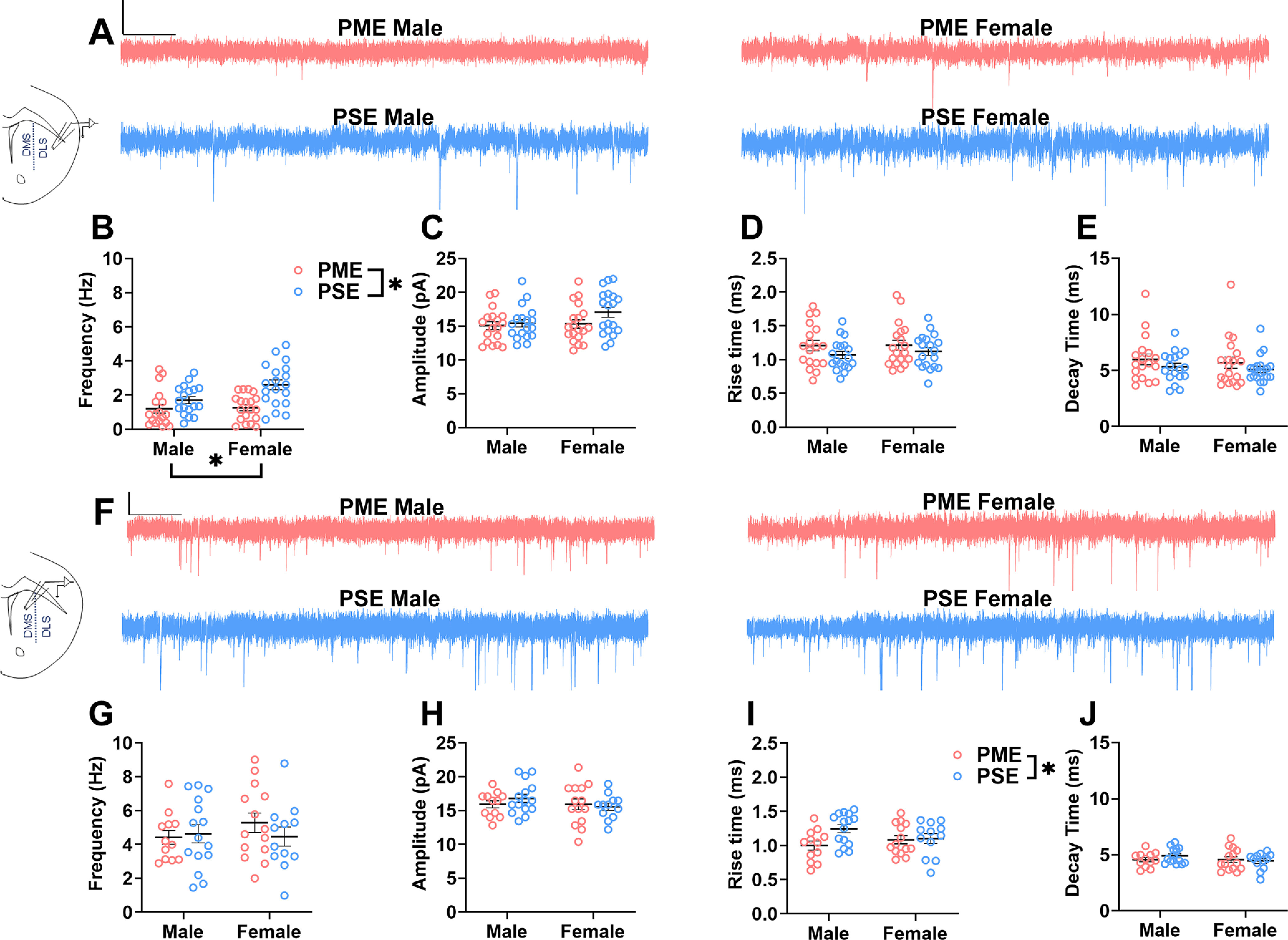
PME impairs medium spiny neuron basal glutamate transmission. ***A***, Representative voltage-clamp traces of sEPSCs from MSNs of the DLS in PME (red) and PSE (blue) male (left) and female (right) adolescent offspring. Calibration: 500 ms, 15 pA. ***B***, In the DLS, PME significantly reduced the frequency of sEPSC events (ANOVA: exposure, *p* = 0.0002). ***C–E***, There was no effect present on amplitude (***C***), rise time (***D***), or decay time (***E***) in the DLS. *n* = 6 PME mice (3 males; 3 females), 37 neurons (18 males; 19 females), and 6 PSE mice (3 males; 3 females), 37 neurons (18 males; 19 females). ***F***, Representative sEPSC traces from MSNs of the DMS in PME (red) and PSE (blue) male (left) and female (right) adolescent offspring. Calibration: 500 ms, 30 pA. ***G***, ***H***, In the DMS, there was not an effect of exposure on either the frequency of events (***G***) or the amplitude of responses (***H***). ***I***, However, PME significantly reduced the rise time of sEPSCs (ANOVA: exposure, *p* = 0.047). ***J***, The decay was not impacted by PME. *n* = 6 PME mice (3 males; 3 females), 27 neurons (12 males; 15 females); and 6 PSE mice (3 males; 3 females), 27 neurons (15 males; 12 females). *p < 0.05.

10.1523/ENEURO.0119-22.2022.f1-4 Figure 1-4 Pathway analysis. This file contains the full KEGG and Reactome pathway analysis results for males and females in both the DLS and DMS. Download Figure 1-4, XLS file.

To further probe differences in the proteome of the dorsal striatum, KEGG (Kyoto Encyclopedia of Genes and Genomes) and Reactome pathway enrichment analyses were performed using g:Profiler on the networks of significant differentially expressed proteins in each dorsal striatum subregion for both sexes (Raudvere et al., 2019). Like the hub protein analysis, very minimal enrichment in any KEGG or Reactome pathways were discovered in the DLS or DMS of females (only two KEGG pathways were enriched in the DMS with zero pathways discovered in the DLS). However, the analyses of males revealed 28 KEGG and 57 Reactome pathways enriched in the DLS and 58 KEGG and 90 Reactome pathways enriched in the DMS using the respective network of differentially expressed proteins (FDR, <0.01; Fig. 1*E*,*F*: pink dots, KEGG pathway terms; blue dots, Reactome pathway terms). The full identity and description of the terms can be found in the supplementary information, but those of particular relevance are highlighted and numbered within Figure 1, *E* and *F*. Of these significantly enriched pathways in the network of differentially abundant proteins in DLS of males (Fig. 1*E*), many were related to neuronal signaling pathways (1, retrograde endocannabinoid signaling, 3, Cyclic guanosine monophosphate (cGMP)–PKG signaling pathway; 5, opioid signaling), synaptic transmission (4, vesicle-mediated transport; 6, transmission across chemical synapses), and neuronal development (7, L1CAM interactions; 8, NGF-stimulated transcription; 9, axon guidance; 10, nervous system development). Similarly, in the DMS of males (Fig. 1*F*), pathways related to neuronal signaling (1, cGMP–PKG signaling pathway; 3, sphingolipid signaling pathway), synaptic transmission (5, cell–cell communication; 6, vesicle-mediated transport; 9, neurexins and neuroligins), and neuronal development (2, 7, axon guidance; 8, nervous system development) were also enriched in the network of differentially abundant proteins.

In the DLS, 5249 phosphopeptides were identified with 156 and 47 differentially expressed (*p* < 0.05) in males and females, respectively (Fig. 2*A*,*B*, left). In the DMS, 5291 phosphopeptides were identified with 77 and 55 differentially expressed (*p* < 0.05) in males and females, respectively (Fig. 2*C*,*D*, left). Many of these proteins were associated with glutamatergic signaling (GluN2B, mGluR7, GRIP1, GLT-1), presynaptic neurotransmitter release [bassoon, Rims1 (regulating synaptic membrane exocytosis protein 1), piccolo], maintaining synaptic architecture (neurofilaments, microtubule associated proteins, ankyrins), ion transport (voltage gated calcium, potassium, and sodium channels), and intracellular synaptic signaling pathways (CaMKII, PKC). Heatmaps of the top 30 differentially expressed phosphorylated proteins were generated to visualize the diversity of phosphorylated proteins identified (Fig. 2*A–D*, middle). Hub proteins for each network of differentially expressed phosphopeptides are displayed on the volcano plots (Fig. 2*A–D*, left) and identified in Table 1. Most of these hub proteins were synapse-associated proteins related to cytoplasmic structure (Sptbn1, Ank2, Ank3, Ank1, Dnm3, Cask, Epb4.1l1, Vcl, Cyfip1) or neuronal signaling pathways (Arrb1, Gapvd1, Dlg2, Camk2b, Arhgef2). To estimate the changes in kinase pathways as a result of the differential phosphopeptide expression, KSEAs were performed (Wiredja et al., 2017). The full enrichment results of the KSEA kinase scores from the differential phosphopeptide expression data of each brain region and sex were then overlaid onto kinome trees using Coral (Metz et al., 2018) to visualize patterns in enrichment among the various kinase families (Extended Data Figs. 2-4, 2-5, 2-6, 2-7). Additionally, the top 30 kinases are presented in Figure 2*A–D*, right, and Table 2. PME led to wide-ranging effects on kinase activity across the DLS and DMS of males and females. Changes in the AGC kinase family including PKC, PKA, PKG, and AKT (PKB), which are well described kinases involved in second-messenger signaling cascades, were frequently observed. PKC and PKA isoforms were identified in the DLS and DMS of both sexes as being significantly dysregulated (FDR, <0.05; Fig. 2*A–D*, right). Like the hub protein analyses, many of the significant kinase scores are involved in pathways that regulate cytoskeletal organization such as Ttbk2, Pak1, Pak2, Pak3, Rock2, Cdc42Bpa, and Cdc42Bpb. Interestingly, females primarily show reduced kinase enrichment in the DLS but increased enrichment in the DMS potentially reflecting subregion-specific shifts in unique phosphorylation pathways.

**Table 2:** Estimated changes in kinase activity from differential phosphopeptide expression networks in the DMS and DLS of males and females (FDR, <0.05)

Gene name	Kinase name	Enrichment *z* score	FDR
DLS of males			
*Cdk9*	Cyclin-dependent kinase 9	6.68	<0.0001
*Ttbk2*	Tau tubulin kinase 2	3.87	0.0039
*Prkca*	Protein kinase C α	3.41	0.0154
DLS of females			
*Pak1*	P21 activated kinase 1	−4.11	0.0003
*Pak2*	P21 activated kinase 2	−4.11	0.0003
*Prkaca*	Protein kinase cAMP-activated catalytic subunit α	−4.11	0.0003
*Clk4*	CDC like kinase 4	−2.71	0.0222
*Prkcd*	Protein kinase C δ	−2.71	0.0222
*Prkci*	Protein kinase C ι	−2.71	0.0222
*Rock2*	Rho associated coiled-coil containing protein kinase 2	−2.71	0.0222
*Prkcz*	Protein kinase C ζ	−2.44	0.0424
DMS of males			
*Cdk9*	Cyclin dependent kinase 9	5.83	<0.0001
*Prkci*	Protein kinase C ι	−4.15	0.0011
*Pak3*	P21 activated kinase 3	−3.58	0.0078
*Pak1*	P21 activated kinase 1	−3.22	0.0217
DMS of females			
*Csnk1E*	Casein kinase 1 ϵ	3.38	0.0083
*Clk2*	CDC like kinase 2	3.47	0.0083
*Mapk11*	Mitogen-activated protein kinase 11	−3.40	0.0083
*Mapk12*	Mitogen-activated protein kinase 12	−3.40	0.0083
*Cdc42Bpa*	CDC42 binding protein kinase α	−3.54	0.0083
*Cdc42Bpb*	CDC42 binding protein kinase β	−3.54	0.0083
*Sgk3*	Serum/glucocorticoid regulated kinase family member 3	3.16	0.0151
*Prkaa2*	Protein kinase AMP-activated catalytic subunit α 2	3.01	0.0221
*Prkci*	Protein kinase C ι	2.90	0.0279
*Cdk9*	Cyclin-dependent kinase 9	2.65	0.0427
*Prkaa1*	Protein kinase AMP-activated catalytic subunit α 1	2.67	0.0427
*Clk1*	CDC like kinase 1	2.72	0.0427
*Mapk13*	Mitogen-activated protein kinase 13	−2.65	0.0427

10.1523/ENEURO.0119-22.2022.f2-1 Figure 2-1 Phosphoproteome DLS. This file contains the full phosphoproteomics results for males and females in the DLS. Download Figure 2-1, XLS file.

10.1523/ENEURO.0119-22.2022.f2-2 Figure 2-2 Phosphoproteome DMS. This file contains the full phosphoproteomics results for males and females in the DMS. Download Figure 2-2, XLS file.

10.1523/ENEURO.0119-22.2022.f2-3 Figure 2-3 KSEA kinase scores. This file contains the full KSEA analysis results and kinase scores for males and females in both the DLS and DMS. Download Figure 2-3, XLS file.

10.1523/ENEURO.0119-22.2022.f2-4 Figure 2-4 Kinome treeplots representing the full KSEA results for males in the DLS. The results from kinase-substrate enrichment analysis were mapped onto kinome treeplots via Coral in which branch color corresponds to significance level, node color corresponds to the *z* score of enrichment, and node size corresponds to the magnitude of enrichment for kinase pathways in the DLS of males. Download Figure 2-4, TIF file.

**Figure 5.**
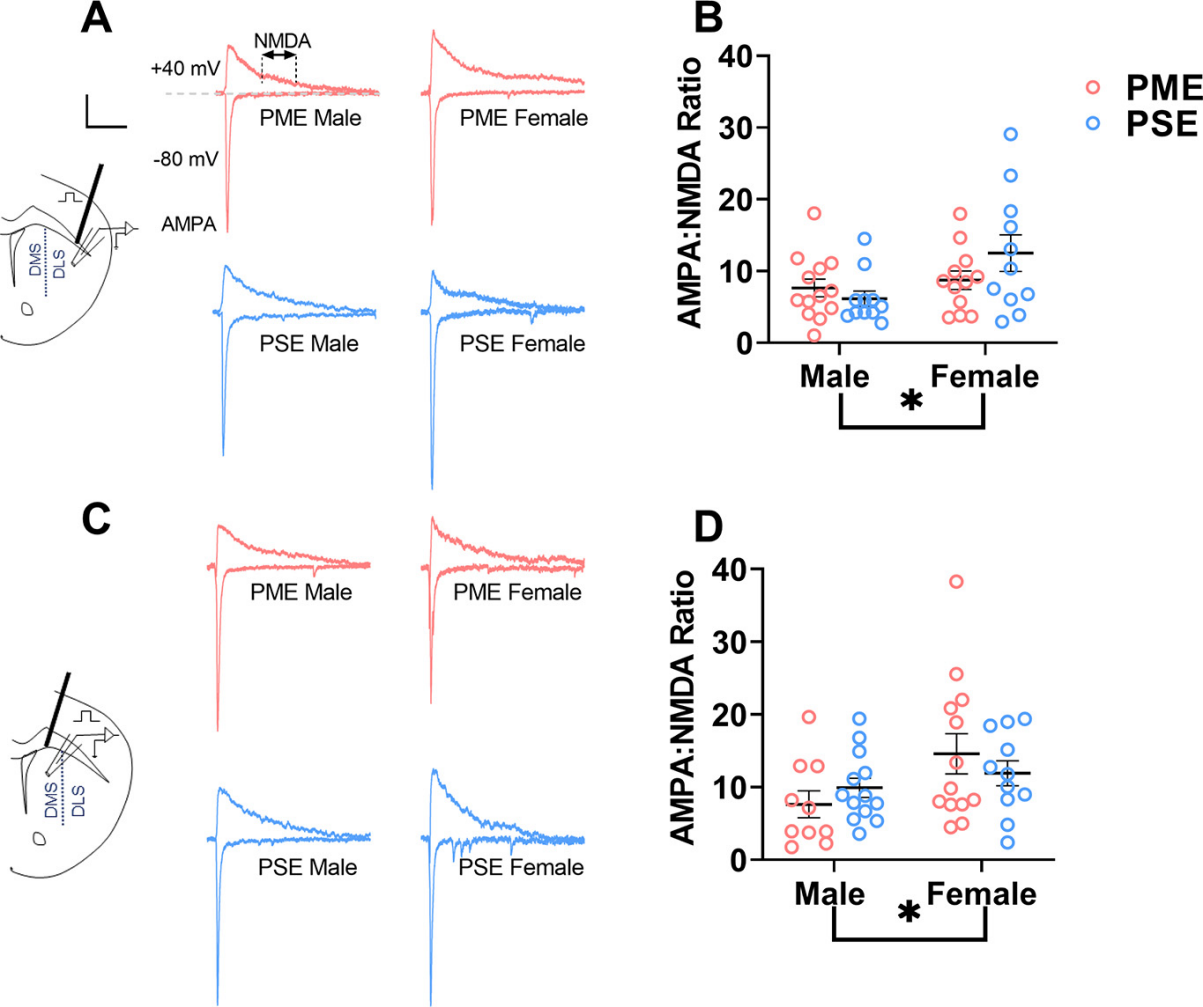
PME effects on AMPA/NMDA currents. ***A***, Representative voltage-clamp traces of AMPA and NMDA current traces from MSNs of the DLS PME (red) and PSE (blue) male (left) and females (right) adolescent offspring. The cell was first held at −80 mV, and AMPAR-mediated EPSCs were electrically evoked. For the NMDAR current, the cell was then held at +40 mV, and an EPSC was again evoked. As the AMPAR component of EPSCs at −80 mV was not apparent 100 ms following the electrical stimulus (i.e., the measured current returned to baseline), the NMDAR-mediated portion of the EPSC at +40 mV was calculated as the average of the measured current over the following 25 ms (i.e., 100–125 ms poststimulus; see dotted lines in ***A*** for the PME-male trace). Calibration: 100 ms, 100 pA. ***B***, In the DLS, a significant sex effect, but no exposure-related effects, was present on AMPA/NMDA current. *n* = 6 PME mice (3 males; 3 females), 25 neurons (13 males; 12 females); and 6 PSE mice (3 males; 3 females), 22 neurons (11 males; 11 females). ***C***, Representative AMPA and NMDA current traces from MSNs of the DMS in PME (red) and PSE (blue) male (left) and female (right) adolescent offspring. Calibration: 100 ms, 100 pA. ***D***, A significant sex effect was also present in the DMS, but no exposure-related effects were present. *n* = 6 PME mice (3 males; 3 females), 23 neurons (10 males; 13 females); and 6 PSE mice (3 males; 3 females), 24 neurons (13 males; 11 females). *p < 0.05.

10.1523/ENEURO.0119-22.2022.f2-5 Figure 2-5 Kinome treeplots representing the full KSEA results for females in the DLS. The results from kinase-substrate enrichment analysis were mapped onto kinome treeplots via Coral in which branch color corresponds to significance level, node color corresponds to the *z* score of enrichment, and node size corresponds to the magnitude of enrichment for kinase pathways in the DLS of females. Download Figure 2-5, TIF file.

**Figure 6.**
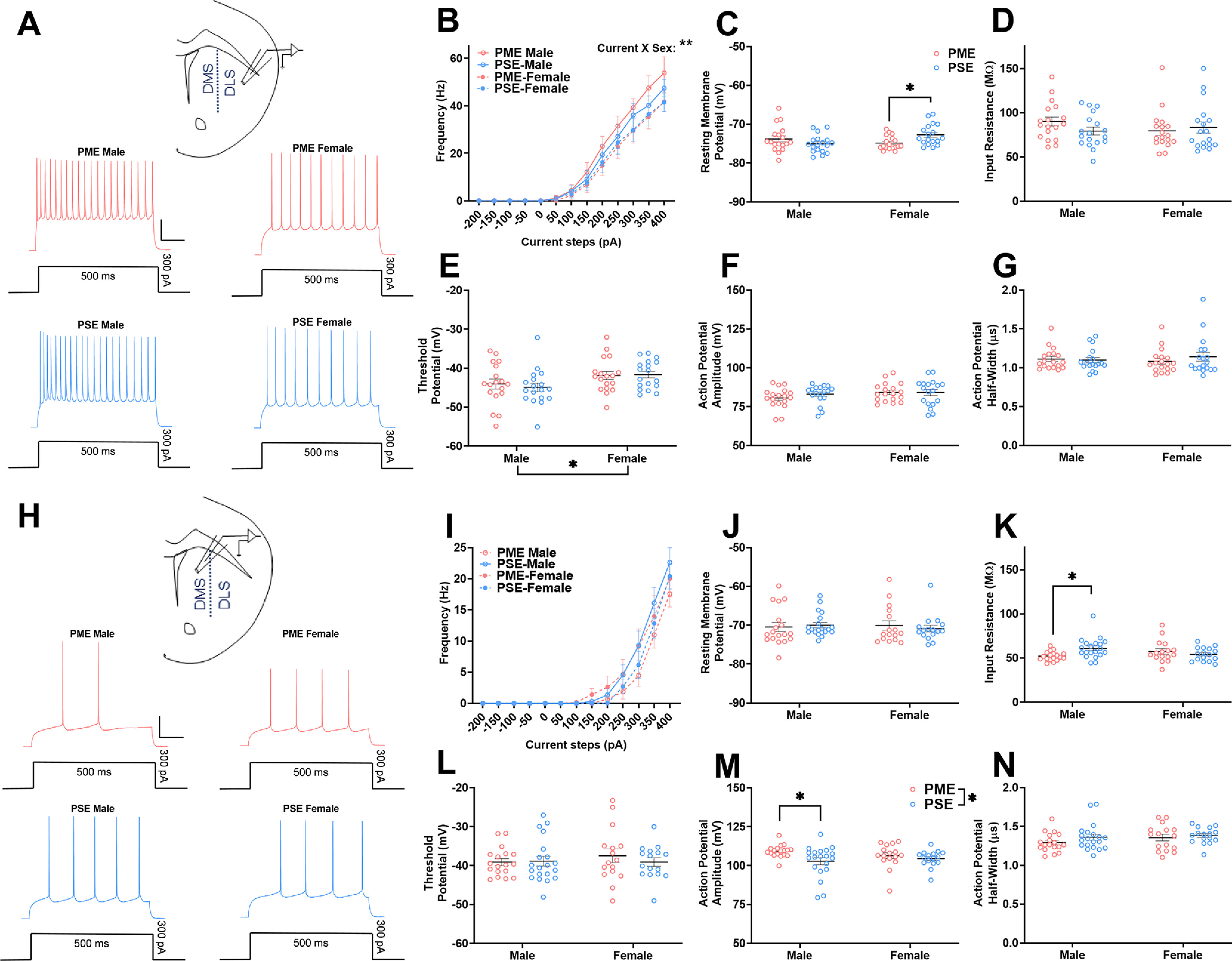
Excitability of dorsal striatal medium spiny neurons is minimally impacted by PME. ***A***, Representative current-clamp traces of action potential firing from MSNs of the DLS in PME (red) and PSE (blue) male (left) and female (right) adolescent offspring. Calibration: 100 ms, 25 mV. ***B***, In the DLS, action potential frequency at various current steps were not significantly different between exposures. ***C***, Female PME MSNs did reveal a slight but significant increase in resting membrane potential compared with PSE-females (ANOVA: interaction, *p* = 0.0062; female PME vs female PSE, *p* = 0.032). ***D–G***, No other exposure related effects were discovered for input resistance (***D***), threshold potential (***E***), peak action potential amplitude (***F***), or action potential half-width (***G***); *n* = 6 PME mice (3 males; 3 females), 36 neurons (18 males; 18 females); and 6 PSE mice (3 males; 3 females), 36 neurons (18 males; 18 females). ***H***, Representative current-clamp traces of action potential firing from MSNs of the DMS in PME (red) and PSE (blue) male (left) and female (right) adolescent offspring. Calibration: 100 ms, 25 mV. ***I***, ***J***, In the DMS, the frequency of action potentials was not affected by sex or exposure (***I***), nor was the resting membrane potential (***J***). ***K***, However, the input resistance was significantly decreased in PME-males compared with PSE-males (ANOVA: exposure × sex, *p* = 0.012; Sidak’s *post hoc* test, *p* = 0.014). ***L***, PME did not alter the threshold potential. ***M***, PME significantly increased the peak action potential amplitude, although this was primarily because of PME-males (ANOVA: exposure, *p* = 0.022; Sidak’s *post hoc* test, *p* = 0.017). ***N***, The action potential half-width was not significantly impacted by PME. *n* = 7 PME mice (4 males; 3 females), 34 neurons (18 males; 16 females); and 6 PSE mice (3 males; 3 females), 36 neurons (20 males; 16 females). *p < 0.05.

10.1523/ENEURO.0119-22.2022.f2-6 Figure 2-6 Kinome treeplots representing the full KSEA results for males in the DMS. The results from kinase-substrate enrichment analysis were mapped onto kinome treeplots via Coral in which branch color corresponds to significance level, node color corresponds to the *z* score of enrichment, and node size corresponds to the magnitude of enrichment for kinase pathways in the DMS of males. Download Figure 2-6, TIF file.

**Figure 7.**
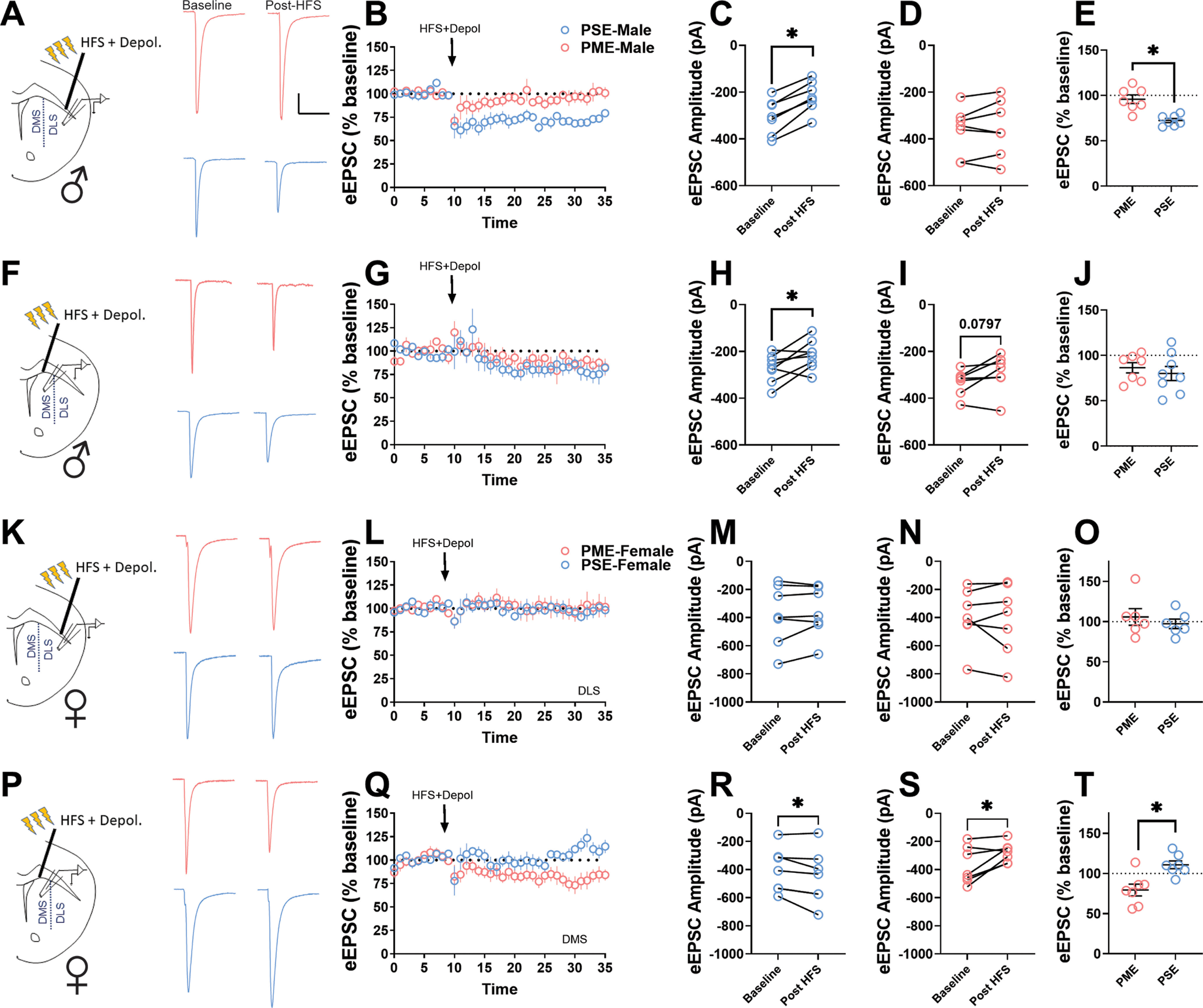
PME ablates endocannabinoid-mediated long-term depression in the DLS of males. ***A***, Representative electrically eEPSC traces from the DLS before and after HFS (three trains of 1 s, 100 Hz stimulation separated by 10 s) plus postsynaptic depolarization (0 mV) in PME (red) and PSE (blue) male mice. Calibration: 50 ms, 100 pA. ***B***, Time series of eEPSC amplitude averages in the DLS over the 35 min recording session. Blue circles, PSE-males; red circles, PME-males. ***C***, ***D***, HFS plus depolarization was capable of significantly reducing the eEPSC amplitude compared with baseline in PSE-males (paired *t* test: *p* < 0.0001; ***C***), but not in PME-males (paired *t* test: *p* = 0.42; ***D***). ***E***, The percentage reduction in eEPSC amplitude during the final 10 min of recording was significantly blunted in PME-males compared with PSE-males (Student’s *t* test, *p* = 0.0007); *n* = 5 PME mice (7 neurons) and 5 PSE mice (7 neurons). ***F***, Representative eEPSC traces from the DMS before and after HFS plus depolarization in PME (red) and PSE (blue) male mice. ***G***, Time series of eEPSC amplitude averages in the DMS over the 35 min recording session. Blue circles, PSE-males; red circles, PME-males. ***H***, ***I***, In the DMS, the protocol reduced eEPSC amplitudes in both PSE-males (paired *t* test: *p* = 0.037; ***H***) and PME-males, although the *p* value did not quite reach the level of significance in PME-males (paired *t* test, *p* = 0.079; ***I***). ***J***, The percentage reduction in eEPSC amplitude during the final 10 min of recording was not significantly different between exposure groups (Student’s *t* test, *p* = 0.53); *n* = 5 PME mice (7 neurons) and 5 PSE mice (8 neurons). ***K***, Representative eEPSC traces from the DLS before and after HFS plus depolarization in PME (red) and PSE (blue) female mice. ***L***, Time series of eEPSC amplitude averages in the DLS over the 35 min recording session. Blue circles, PSE-females; red circles, PME-females. ***M***, ***N***, HFS plus depolarization did not impact eEPSC amplitudes in either PSE-females (paired *t* test, *p* = 0.31; ***M***) or PME-females (paired *t* test, *p* = 0.69; ***N***). ***O***, The percentage reduction in eEPSC amplitude did not differ between exposure groups (Student’s *t* test, *p* = 0.49); *n* = 6 PME mice (6 neurons) and 6 PSE mice (6 neurons). ***P***, Representative eEPSC traces from the DMS before and after HFS plus depolarization in PME (red) and PSE (blue) female mice. ***Q***, Time series of eEPSC amplitude averages in the DMS over the 35 min recording session. Blue circles, PSE-females; red circles, PME-females. ***R***, HFS plus depolarization produced a mild potentiation in PSE-females as eEPSC amplitudes increased (paired *t* test, *p* = 0.046). ***S***, However, PME-females exhibited a significant reduction in eEPSC from baseline (paired *t* test, *p* = 0.032). ***T***, The percentage change in eEPSC amplitude was significantly different in the DMS between exposure groups (Student’s *t* test, *p* = 0.0037); *n* = 6 PME mice (7 neurons) and 6 PSE mice (7 neurons). *p < 0.05.

10.1523/ENEURO.0119-22.2022.f2-7 Figure 2-7 Kinome treeplots representing the full KSEA results for females in the DMS. The results from kinase-substrate enrichment analysis were mapped onto kinome treeplots via Coral in which branch color corresponds to significance level, node color corresponds to the *z* score of enrichment, and node size corresponds to the magnitude of enrichment for kinase pathways in the DMS of females. Download Figure 2-7, TIF file.

Interestingly, there was little overlap in the differentially abundant proteins and phosphopeptides in the DLS and DMS (Fig. 3). In the DLS, only one protein was identified in both analyses, and this was the membrane cytoskeleton-associated protein α-adducin, which exhibited reduced abundance in PME-males but an increase in one phosphopeptide abundance, suggesting the increased phosphorylation of this protein may result in reduced expression, or vice versa (Fig. 3*A*). In the DMS, transformer-2 protein homolog α (Tra2a), which is involved with mRNA splicing was found to be increased in abundance but revealed one decreased phosphopeptide change in PME-males (Fig. 3*C*). There were also several proteins that demonstrated both increased and decreased phosphopeptide abundance in the DLS (the potassium channel, K_v_7.2, PKCγ, microtubule-associated protein 1B, adenylate cyclase (type 9), CaMKIIα, and the cAMP-dependent protein kinase type II-β) and DMS (bassoon and the proton myoinositol cotransporter) of males. Nonetheless, there was no overlap in differentially abundant proteins and phosphopeptides in females, either increased or decreased in abundance (Fig. 3*B*,*D*). The surprising lack of overlap between significant proteins and phosphopeptides indicates that the phosphopeptide changes described herein are not attributable to global protein abundance changes, but, instead, the changes in phosphopeptide expression results from dynamic phosphomodulation of proteins regardless of global abundance. Overall, these multiomic data indicate that PME induces persistent and widespread changes that are unique to DLS and DMS proteomes and phosphoproteomes with many effects associated with proteins and processes related to synaptic transmission and signaling.

### Medium spiny neuron excitatory transmission

The MSNs of the dorsal striatum integrate glutamatergic input from the cortex, thalamus, and amygdala regions to ultimately facilitate motor behavior, with many of these circuits relevant to the pathologic consumption of alcohol, and may contribute to the alcohol reward phenotype we have previously reported in these mice (Tepper et al., 2007; Peak et al., 2019; Grecco et al., 2022). Given the numerous changes in proteins and phosphopeptides related to glutamatergic signaling and neurotransmitter release (GluN2B, GRIN1, mGluR7, GRIP1, GLT-1, bassoon, Rims1, piccolo), we next examined spontaneous glutamate-mediated currents and AMPA/NMDA current ratios to evaluate excitatory input to MSNs in the DLS and DMS.

The frequencies in the DLS were significantly reduced (ANOVA: exposure, *F*_(1,70)_ = 15.4, *p* = 0.0002; sex, *F*_(1,70)_ = 4.17, *p* = 0.045; interaction, *F*_(1,70)_ = 3.13, *p* = 0.081; Fig. 4*B*), indicating reduced basal excitatory input onto MSNs in the DLS of adolescent PME offspring. No exposure-related effects on sEPSC amplitude (ANOVA: exposure, *F*_(1,70)_ = 2.66, *p* = 0.11; sex, *F*_(1,70)_ = 2.10, *p* = 0.15; interaction, *F*_(1,70)_ = 1.12, *p* = 0.29), rise time (ANOVA: exposure, *F*_(1,70)_ = 3.053, *p* = 0.085; sex, *F*_(1,70)_ = 0.20, *p* = 0.66; interaction, *F*_(1,70)_ = 0.138, *p* = 0.71), or decay (ANOVA: exposure, *F*_(1,70)_ = 2.43, *p* = 0.12; sex, *F*_(1,70)_ = 0.40, *p* = 0.53; interaction, *F*_(1,70)_ = 0.0065, *p* = 0.93) were discovered in the DLS (Fig. 4*C–E*). In the DMS, there were no sex- or exposure-related effects on the frequencies of sEPSCs (ANOVA: exposure, *F*_(1,49)_ = 0.307, *p* = 0.58; sex, *F*_(1,49)_ = 0.416, *p* = 0.52; interaction, *F*_(1,49)_ = 0.902, *p* = 0.35; Fig. 4*G*) or the amplitudes of sEPSCs (ANOVA: exposure, *F*_(1,49)_ = 0.173, *p* = 0.68; sex, *F*_(1,49)_ = 0.936, *p* = 0.34; interaction, *F*_(1,49)_ = 0.915, *p* = 0.34; Fig. 4*H*). There was a significant effect of PME on DMS sEPSC rise times (ANOVA: exposure, *F*_(1,49)_ = 4.15, *p* = 0.047; sex, *F*_(1,49)_ = 0.203, *p* = 0.65; interaction, *F*_(1,49)_ = 3.09, *p* = 0.09; Fig. 4*I*) with PME offspring exhibiting significantly shorter rise times. There was no exposure effect on DMS sEPSC decay times (ANOVA: exposure, *F*_(1,49)_ = 0.245, *p* = 0.62; sex, *F*_(1,49)_ = 1.16, *p* = 0.29; interaction, *F*_(1,49)_ = 1.18, *p* = 0.28; Fig. 4*J*).

In the DLS, there was a significant effect of sex, but not exposure on AMPA/NMDA current ratios (ANOVA: exposure, *F*_(1,43)_ = 0.511, *p* = 0.48; sex, *F*_(1,43)_ = 5.43, *p* = 0.025; interaction, *F*_(1,43)_ = 2.69, *p* = 0.11; Fig. 5*B*). Similarly, in the DMS, there was a significant effect of sex but not exposure on AMPA/NMDA current ratios (ANOVA: exposure, *F*_(1,43)_ = 0.010, *p* = 0.92; sex, *F*_(1,43)_ = 4.79, *p* = 0.034; interaction, *F*_(1,43)_ = 1.46, *p* = 0.23; Fig. 5*D*).

### Medium spiny neurons excitability

As we observed several changes the abundance and phosphorylation status of ion channels that could impact neuronal excitability (e.g., Na_v_1.6, Na_v_1.3, Na_v_2.1, K_V_2.1, and K_v_7.2), we next examined MSN excitability. PME produced few effects on MSN excitability in the dorsal striatum. In the DLS, action potential frequencies were not significantly different between exposure groups [repeated-measures ANOVA (rmANOVA): exposure, *F*_(1,68)_ = 0.175, *p* = 0.68; current, *F*_(2.35,159.6)_ = 195.2, *p* < 0.0001; sex, *F*_(1,68)_ = 2.56, *p* = 0.11; current × exposure, *F*_(8,544)_ = 0.282, *p* = 0.97; current × sex, *F*_(8,544)_ = 1.85, *p* = 0.065; exposure × sex, *F*_(1,68)_ = 0.465, *p* = 0.50; current × exposure × sex, *F*_(8,544)_ = 0.305, *p* = 0.96; Fig. 6*B*]. Female PME MSNs did reveal a slight but significant increase in RMPs (ANOVA: exposure, *F*_(1,68)_ = 0.453, *p* = 0.50; sex, *F*_(1,68)_ = 1.12, *p* = 0.29; interaction, *F*_(1,68)_ = 7.98, *p* = 0.0062; Sidak’s *post hoc* test: PME-female vs PSE-female, *p* = 0.032; Fig. 6*C*). However, no other exposure-related effects were discovered for input resistances (ANOVA: exposure, *F*_(1,68)_ = 0.440, *p* = 0.51; sex, *F*_(1,68)_ = 0.389, *p* = 0.54; interaction, *F*_(1,68)_ = 1.86, *p* = 0.18; Fig. 6*D*), threshold potentials (ANOVA: exposure, *F*_(1,68)_ = 0.0838, *p* = 0.77; sex, *F*_(1,68)_ = 6.43, *p* = 0.014; interaction, *F*_(1,68)_ = 0.263, *p* = 0.61; Fig. 6*E*), peak action potential amplitudes (ANOVA: exposure, *F*_(1,68)_ = 0.484, *p* = 0.49; sex, *F*_(1,68)_ = 2.04, *p* = 0.16; interaction, *F*_(1,68)_ = 0.621, *p* = 0.43; Fig. 6*F*), or action potential half-widths (ANOVA: exposure, *F*_(1,68)_ = 0.293, *p* = 0.59; sex, *F*_(1,68)_ = 0.0339, *p* = 0.85; interaction, *F*_(1,68)_ = 0.783, *p* = 0.38; Fig. 6*G*).

In the DMS, MSNs fired fewer action potentials in response to injected currents compared with DLS MSNs. There were no significant effects of sex or current on action potential frequencies (rmANOVA: exposure, *F*_(1,66)_ = 0.214, *p* = 0.65; current, *F*_(1.46,96.3)_ = 142.8, *p* < 0.0001; sex, *F*_(1,66)_ = 0.053, *p* = 0.82; current × exposure, *F*_(8,528)_ = 0.966, *p* = 0.46; current × sex, *F*_(8,528)_ = 0.063, *p* = 0.99; exposure × sex, *F*_(1,66)_ = 2.29, *p* = 0.13; current × exposure × sex, *F*_(8,528)_ = 1.42, *p* = 0.18; Fig. 6*I*). PME did not impact RMPs in the DMS (ANOVA: exposure, *F*_(1,66)_ = 0.030, *p* = 0.86; sex, *F*_(1,66)_ = 0.062, *p* = 0.80; interaction, *F*_(1,66)_ = 0.398, *p* = 0.53; Fig. 6*J*). However, there was a sex-dependent effect of PME on input resistances (ANOVA: exposure, *F*_(1,66)_ = 1.41, *p* = 0.24; sex, *F*_(1,66)_ = 0.096, *p* = 0.76; interaction, *F*_(1,66)_ = 6.72, *p* = 0.012; Sidak’s *post hoc* test: PME-male vs PSE-male, *p* = 0.014; Fig. 6*K*), indicating there was a smaller change in membrane voltages in response to injected currents in PME-male MSNs of the DMS. There were no significant effects on threshold potentials (ANOVA: exposure, *F*_(1,66)_ = 0.290, *p* = 0.59; sex, *F*_(1,66)_ = 0.289, *p* = 0.59; interaction, *F*_(1,66)_ = 0.534, *p* = 0.47; Fig. 6*L*). There was a main effect of exposure on peak action potential amplitudes, and, although the interaction did not reach the level of significance, these exposure effects appear to be driven by PME-males (ANOVA: exposure, *F*_(1,66)_ = 5.51, *p* = 0.022; sex, *F*_(1,66)_ = 0.119, *p* = 0.73; interaction, *F*_(1,66)_ = 1.72, *p* = 0.19; Sidak’s *post hoc* test: PME-male vs PSE-male, *p* = 0.017; Fig. 6*M*). PME did not significantly impact action potential half-widths (ANOVA: exposure, *F*_(1,66)_ = 1.80, *p* = 0.18; sex, *F*_(1,66)_ = 1.43, *p* = 0.24; interaction, *F*_(1,66)_ = 0.379, *p* = 0.54; Fig. 6*N*).

### Endocannabinoid-mediated long-term depression

As the KEGG pathways analysis in the DLS of males revealed that retrograde endocannabinoid signaling was differentially enriched [as a result of protein/phosphorylation changes to the L-type calcium channel, Ca_V_1.2, various α- and β-subunits of G-proteins known to interact with the cannabinoid receptor 1 (CB1) and Rims1], we next tested the expression of eCB-LTD in the DLS and DMS as this signaling pathway has been shown to be disrupted by drugs of abuse in adult rodents (Adermark et al., 2011; Nazzaro et al., 2012; DePoy et al., 2013; Atwood et al., 2014; Abburi et al., 2016). The time series of electrically evoked EPSC (eEPSC) amplitude averages over the 35 min recording session is provided in Figure 7, *B* and *G*, for the DLS and DMS, respectively. HFS plus depolarization produced a sustained reduction in the eEPSC amplitude compared with baseline in the DLS of PSE-males (paired *t* test: *t*_(6)_ = 9.13, *p* < 0.0001; Fig. 7*C*). However, this same protocol did not significantly impact the eEPSC amplitude in PME-males (paired *t* test: *t*_(6)_ = 0.874, *p* = 0.42; Fig. 7*D*), indicating that eCB-LTD is ablated in the DLS of PME-males. Furthermore, the percentage reduction in eEPSC amplitude during the final 10 min of recording was significantly blunted in PME-males compared with PSE-males (Student’s *t* test: *t*_(12)_ = 4.52, *p* = 0.0007; Fig. 7*E*). In the DMS, the LTD was more variable, but the stimulation reduced the eEPSC baseline in PSE-males (paired *t* test: *t*_(7)_ = 2.58, *p* = 0.037; Fig. 7*H*). In PME-males, this sustained reduction in eEPSC baseline did not quite reach the level of significance (paired *t* test: *t*_(6)_ = 2.11, *p* = 0.079; Fig. 7*I*). Importantly, the average change in amplitude from baseline did not differ between exposure groups (paired *t* test: *t*_(13)_ = 0.645, *p* = 0.53; Fig. 7*J*) supporting the idea that differential expression of eCB-LTD in the dorsal striatum of PME-males is unique to the DLS, as the proteomics studies revealed.

Although the retrograde endocannabinoid signaling pathway was not enriched in females for either subregion of the dorsal striatum, we sought to test eCB-LTD in females as a comparison for the findings in males. The stimulation did not produce LTD in either PSE-females (paired *t* test: *t*_(6)_ = 1.10, *p* = 0.31; Fig. 7*M*) or PME-females (paired *t* test: *t*_(6)_ = 0.414, *p* = 0.69; Fig. 7*N*). The percentage reduction in eEPSC amplitude also did not significantly differ between exposure groups (Student’s *t* test: *t*_(10)_ = 0.712, *p* = 0.49; Fig. 7*O*). Surprisingly, HFS plus depolarization produced an increase in eEPSC amplitudes in PSE-females (paired *t* test: *t*_(6)_ = 2.51, *p* = 0.046; Fig. 7*R*), while eCB-LTD was exhibited in PME-females (paired *t* test: *t*_(6)_ = 2.78, *p* = 0.032; Fig. 7*S*). The percentage change in eEPSC amplitude was significantly different in the DMS between female exposure groups (Student’s *t* test: *t*_(12)_ = 3.60, *p* = 0.0037; Fig. 7*T*).

**Figure 8.**
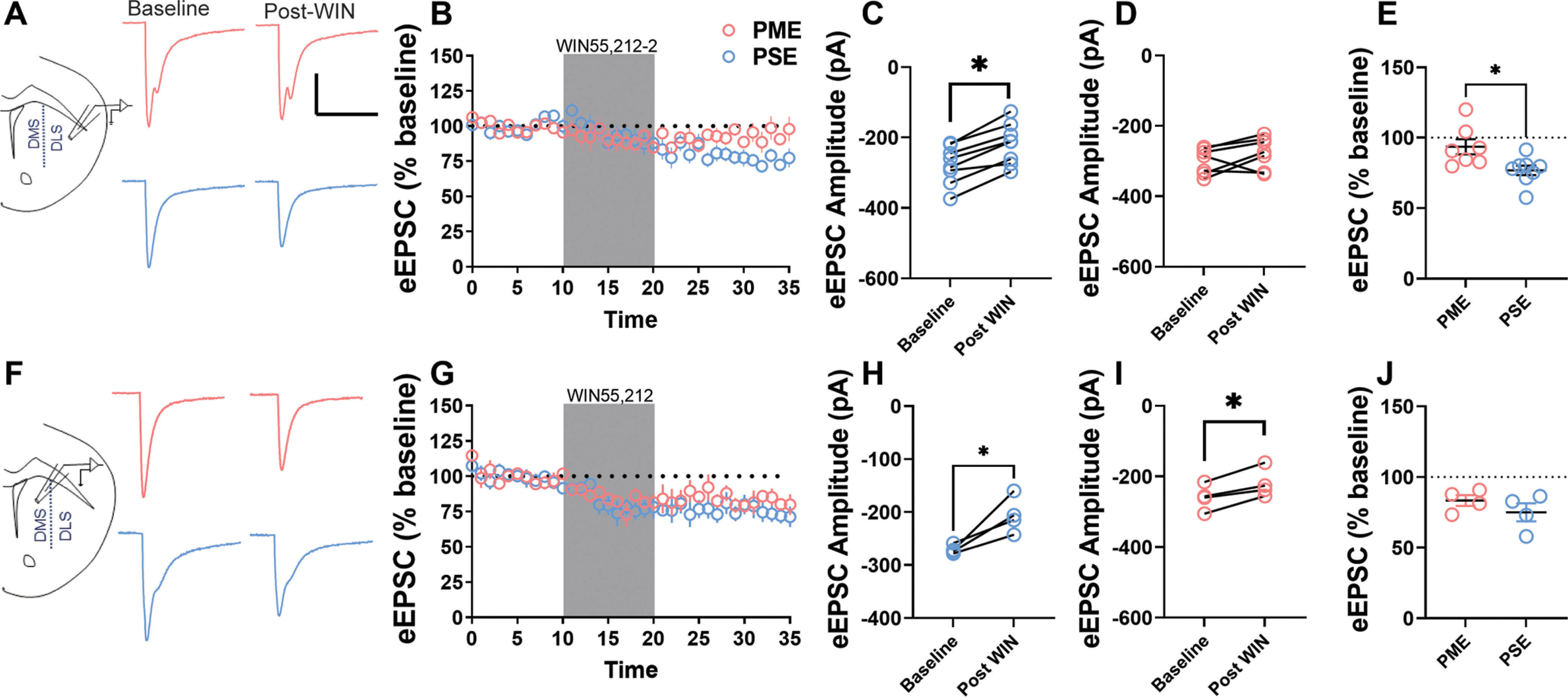
Direct activation CB1 receptors does not rescue endocannabinoid-mediated long-term depression in the DLS of PME-males. ***A***, Representative eEPSC traces from the DLS before and after WIN55,212-2 (1 μm, 10 min) application in PME (red) and PSE (blue) male mice. Calibration: 50 ms, 100 pA. ***B***, Time series of electrically eEPSC amplitude averages in the DLS over the 35 min recording session. Blue circles, PSE-males; red circles, PME-males. ***C***, ***D***, Application of the CB1 agonist WIN55,212-2 (1 μm) significantly reduced the eEPSC amplitude compared with baseline in PSE-males (paired *t* test, *p* = 0.0001; ***C***), but not in PME-males (paired *t* test, *p* = 0.18; ***D***). ***E***, The percentage reduction in eEPSC amplitude during the final 10 min of recording was significantly different between exposure groups (Student’s *t* test, *p* = 0.018); *n* = 3 PME mice (8 neurons) and 3 PSE mice (8 neurons). ***F***, Representative eEPSC traces from the DMS before and after WIN55,212-2-2 (1 μm, 10 min) application in PME (red) and PSE (blue) mice. ***G***, Time series of eEPSC averages in the DMS over the 35 min recording session. Blue circles, PSE-males; red circles, PME-males. ***H***, ***I***, Application of the CB1 agonist WIN55,212-2 significantly reduced the eEPSC amplitude compared with baseline in both PSE-males (paired *t* test, *p* = 0.032; ***H***) and in PME-males (paired *t* test, *p* = 0.015; ***I***). ***J***, The percentage reduction in eEPSC amplitude did not differ between PME-males and PSE-males (Student’s *t* test, *p* = 0.31); *n* = 2 PME mice (4 neurons) and 2 PSE mice (4 neurons). *p < 0.05.

We next hypothesized that bypassing endogenous endocannabinoid production using the CB1 agonist WIN55,212-2 could rescue the disrupted retrograde endocannabinoid signaling in the male DLS supported by our proteomic and electrophysiology studies. In PSE-males, bath application of a CB1 receptor agonist, WIN55,212-2, induced a long-lasting reduction in eEPSC amplitude in the DLS (Fig. 8*B*); however, WIN55,212-2 did not produce LTD in PME-males (Fig. 8*B*). Paired *t* tests indicated that this reduction of eEPSC amplitudes was significant in PSE-males (paired *t* test: *t*_(7)_ = 7.55, *p* = 0.0001; Fig. 8*C*), but not in PME-males (paired *t* test: *t*_(6)_ = 1.50, *p* = 0.18; Fig. 8*D*). Further, the average change in eEPSC amplitude from baseline during the final 10 min of the recording was significantly diminished in PME-males compared with PSE-males (Student’s *t* test: *t*_(13)_ = 2.71, *p* = 0.018; Fig. 8*E*). Similar to the results of our HFS stimulation and proteomic studies, this CB1-mediated LTD remained intact for both PSE- and PME-males in the DMS, further underscoring the subregion specificity of our plasticity findings (Fig. 8*F*,*G*, for representative eEPSC traces and time series data from the DMS before and after WIN55,212-2 application). WIN55,212-2 application reduced eEPSC amplitudes in both PSE-males (paired *t* test: *t*_(3)_ = 3.78, *p* = 0.032; Fig. 8*H*) and PME-males (paired *t* test: *t*_(3)_ = 5.03, *p* = 0.015; Fig. 8*I*). Additionally, there were no significant effects of exposure on the average change in eEPSC from baseline during the final 10 min of recording (Student’s *t* test: *t*_(6)_ = 1.12, *p* = 0.31; Fig. 8*J*). These findings suggest that the disruption in endocannabinoid signaling in the DLS of PME-males may result from disrupted CB1-mediated signaling at the presynaptic terminal.

## Discussion

These findings demonstrate that prenatal opioid exposure produces neuroadaptations in the dorsal striatum that persist at least through to adolescence. The impact of the proteome, phosphoproteome, synaptic plasticity, glutamatergic transmission, and excitability likely alters the functional output of the dorsal striatum. Consequently, these alterations may contribute to the aberrant behavioral development and altered reward phenotype that we previously described in PME offspring (Grecco et al., 2021a, 2022). This study advances the field of prenatal opioid exposure and opioid physiology in several ways. First, while researchers have performed studies using animal models of prenatal opioid exposure for decades (Byrnes and Vassoler, 2018), the majority of these studies have emphasized the developmental and behavioral characterization of these models with minimal mechanistic insights. Using a combined multiomics and electrophysiological approach, this investigation provides several mechanistic insights into how opioid exposure may disrupt neuronal functioning and behavior. Next, although the opioid system and endocannabinoid system have long been known to be implicated in addiction, cross talk between opioid and endocannabinoid signaling has been a more recent area of investigation (López-Moreno et al., 2010). Our eCB-LTD assessment indicates that exogenous opioids can disrupt endocannabinoid signaling, further supporting the functional overlap between the opioid and cannabinoid system that is heavily implicated in the pathophysiology of various psychiatric illnesses. Last, we and others have shown that opioid (Atwood et al., 2014), alcohol (Adermark et al., 2011; DePoy et al., 2013), nicotine (Abburi et al., 2016), and 9-tetrahydrocannabinol (THC; Nazzaro et al., 2012) treatment to adolescent or adult rodents all impair eCB-LTD in the DLS. This is the first study, to our knowledge, to demonstrate that maternal administration of a rewarding drug can impair eCB-LTD in the DLS of offspring that persists to adolescence.

The disruption in eCB-LTD in the DLS of PME-males is quite intriguing. eCB-LTD represents a form of inhibitory plasticity that reduces glutamate transmission from various cortical inputs to the dorsal striatum. We identified several protein changes known to be involved in the activation and expression of eCB-LTD unique to the DLS of PME-males. On the postsynaptic side, our multiomics analysis revealed a reduction in the phosphorylated L-type calcium channel Ca_V_1.2 in PME-males, suggesting the dendritic calcium-dependent release of endogenous endocannabinoids may be impaired in PME-males, preventing the induction of eCB-LTD. However, LTD induced by both stimulation and an exogenous CB1 agonist was disrupted, indicating the disruption in eCB-LTD in PME-males most likely occurs presynaptically. On the presynaptic side, the α- and β-subunits of G-proteins known to interact with the CB1 receptor were increased in PME-males, indicating that downstream signaling of CB1 receptor activation could be disrupted. Additionally, both adenylate cyclase (type 9) and Rims1 displayed differential phosphopeptide expression in the phosphoproteome, and these proteins have been shown to regulate eCB-LTD in the hippocampus (Chevaleyre et al., 2007). The presynaptic cAMP/PKA pathway downstream of CB1 receptor activation culminates in enduring changes to the phosphorylation status of release machinery proteins such as Rims1. Therefore, further mechanistic work on exactly how PME induces this loss of eCB-LTD may start by investigating downstream signaling of the CB1 receptor such as the presynaptic cAMP/PKA pathway. The combination of these protein abundance level alterations and phosphorylation changes may contribute to the disruption in endocannabinoid signaling described herein. Importantly, our omics results did not reveal changes in the dopamine receptors or transporters, suggesting that the impact on LTD may be independent of dopaminergic signaling.

Cortical, amygdalar, and thalamic inputs converge onto the MSNs of the dorsal striatum, which ultimately regulate basal ganglia output to control the learning and performance of instrumental actions (Peak et al., 2019). Therefore, the neuroadaptations described herein may have particular relevance to the altered addiction-related behaviors observed in prenatal opioid-exposed animals (Grecco and Atwood, 2020). Interestingly, the KEGG pathway analysis of the differential proteome in the DLS of males revealed an enrichment in the alcoholism pathway (Fig. 1*E*, labeled 2). We have recently discovered that adolescent PME-males drink significantly more alcohol relative to PSE-males and exhibit quinine-resistant alcohol drinking, a characteristic that may represent compulsive alcohol use (Hopf and Lesscher, 2014). Disruption in eCB-LTD in the DLS shifts behavior from goal oriented to habitual. For instance, Nazzaro et al. (2012) demonstrated that 5 d of THC treatment abolished eCB-LTD in the DLS (but not the DMS), and accelerated habit learning. Additionally, studies by DePoy et al. (2013, 2015) revealed that chronic intermittent alcohol vapor exposure diminished eCB-LTD in the DLS and disrupted cue-induced responding for a food reward. These studies suggest that normal eCB-LTD in the DLS is important for maintaining the balance between habitual and goal-oriented behavioral strategies and that disruptions in this endocannabinoid signaling may shift this balance toward more habitual and compulsive behavioral responses. Therefore, the disruption in eCB-LTD in the DLS of PME-males may contribute to the alcohol drinking phenotype previously characterized (Grecco et al., 2022), but further mechanistic studies will be required to determine whether restoring eCB-LTD rescues the alcohol-drinking phenotype in PME-males.

Glutamatergic signaling may contribute to the psychopathology induced by prenatal opioid exposure. Other models of prenatal opioid exposure revealed altered glutamate receptor expression (Yang et al., 2006; Lin et al., 2009; Wu et al., 2018) and measures of glutamatergic transmission (Yang et al., 2000, 2003); however, these studies were primarily localized to the hippocampus. Coadministration of dextromethorphan, an NMDA receptor antagonist, alongside PME was sufficient to prevent the increased conditioned place preference to methadone in PME rats later in life (Chiang et al., 2015). In general, a drug-induced imbalance in glutamate signaling within the striatum is thought to underlie the neuropathophysiology associated with commonly misused drugs, including opioids (Kalivas, 2009; Kalivas and Volkow, 2011; Hearing et al., 2018). In conjunction with the role of eCB-LTD, AMPA receptors in DLS are also known to contribute to habit learning (Corbit et al., 2014). Therefore, PME-induced changes in DLS glutamate signaling may also contribute to our previously observed phenotypes (Grecco et al., 2021a, 2022). In line with this rationale, we found that presynaptic glutamate release was reduced in the DLS of both male and female PME offspring. There were several differences in the phosphorylation status of presynaptic architectural proteins in both PME-males and PME-females such as the microtubule-associated protein 1B (Map1b), neurofilament heavy polypeptide (Nefh), DmX-like protein 2 (Dmxl2), and ankyrin-2 (Ank2). In particular, both Map1b and Ank2 have been shown to impact presynaptic glutamate release (Bodaleo et al., 2016; Lippi et al., 2016). Therefore, the impact of PME on the phosphorylation state of Map1b and Ank2 could lead to the shared reduction in glutamate release in both males and females. Conversely, the kinetics of glutamate transmission were altered in the DMS of PME offspring with both sexes exhibiting faster excitatory current kinetics, which could reflect changes in the composition of AMPA or NMDA receptors at the membrane, which may not be easily assessed by our omics analysis. Paradoxically, PME prevents endocannabinoid-driven inhibitory plasticity of glutamate transmission in the DLS, yet PME offspring demonstrate reduced basal glutamate release. This combination of findings may represent occlusion whereby DLS synapses in PME offspring are already undergoing maximal eCB-LTD, and our LTD protocol was not able to reduce eEPSC amplitude further (akin to a floor effect). Indeed, other rewarding substances such as cocaine (Zlebnik and Cheer, 2016) and THC (Friend et al., 2017) have been shown to occlude eCB-LTD in other brain regions. Further work will be necessary to investigate these possibilities.

Our multiomic analysis revealed wide-ranging differences in protein abundance, phosphorylation patterns, and enriched KEGG, Reactome, and kinase pathways. The greater effect in males on the dorsal striatal proteome is surprising, but it remains difficult to speculate at this time on why this sex effect may be present. As discussed above, this greater effect on the DLS proteome in males may contribute to the altered alcohol drinking patterns in PME-males relative to PSE-males, which aligns with the lack of alcohol drinking differences in females (Grecco et al., 2022). However, there may be other brain regions where the proteomic effects may be larger in PME-females. For instance, PME-females demonstrate a prominent hyperlocomotion in response to repeated alcohol injections not seen in PME-males (Grecco et al., 2022). Drug-induced hyperactivity is canonically associated with dopaminergic signaling between the ventral tegmental area (VTA) and nucleus accumbens; therefore, one may predict greater proteomic effects in the VTA or accumbens, although more studies are needed to assess this.

There are several limitations to the proteomics and phosphoproteomics analysis. We dissected DLS and DMS bulk tissue; therefore, the protein/phosphopeptide level differences observed may result from changes in the MSNs, interneurons, glial cells, and/or presynaptic inputs from regions projecting into the dorsal striatum. While we used sample sizes in line with prior publications (Sharma et al., 2015; Kohtala et al., 2016; Boza-Serrano et al., 2018; Grecco et al., 2021b) and quantified a relatively high number of total proteins (>8000 and >9000 proteins in the DLS and DMS, respectively), a larger sample size may have uncovered additional differentially expressed proteins/phosphopeptides, particularly for lowly expressed proteins. Despite these limitations, we encourage readers to carefully examine our full multiomic datasets for questions that may be of interest to them. Regarding our electrophysiological assessments, future studies will need to distinguish whether these neuroadaptations are localized to specific striatal circuits (e.g., corticostriatal, amygdalostriatal, or thalamostriatal) and whether they exhibit cell-type specificity (D_1_ vs D_2_ expressing MSNs). The lack of eCB-LTD in the DLS for either female exposure group and apparent LTP in PSE-females was rather surprising; however, few studies, to our knowledge, have investigated the sex-dependent effects of eCB-LTD in the dorsal striatum. While a further assessment in the females is outside the scope of the current work, future work will be required to investigate these interesting differences in plasticity. Future studies will need to examine inter-animal or interlitter susceptibility to PME. For instance, one PME-male has a proteomic profile that looks different from others in the group, but it remains difficult at this time to fully explain this unique result. Finally, we should acknowledge that the findings herein should not discourage the use of methadone in pregnant women, as overwhelming evidence indicates it is beneficial for the treatment of OUD. Nonetheless, this study provides compelling evidence that prenatal opioid exposure produces long-lasting neuroadaptations in the dorsal striatum that disrupts glutamatergic and endocannabinoid signaling, which may have important implications for habit learning and compulsive drug and alcohol seeking and consumption.

## Synthesis

Reviewing Editor: Alexxai Kravitz, Washington University in St. Louis Decisions are customarily a result of the Reviewing Editor and the peer reviewers coming together and discussing their recommendations until a consensus is reached. When revisions are invited, a fact-based synthesis statement explaining their decision and outlining what is needed to prepare a revision will be listed below. The following reviewer(s) agreed to reveal their identity: NONE. Note: If this manuscript was transferred from JNeurosci and a decision was made to accept the manuscript without peer review, a brief statement to this effect will instead be what is listed below.

Congrats! And thanks for your responsive revisions and clear rebuttal statement
