# Supplementary figures and images for: Prenatal Opioid Exposure Impairs Endocannabinoid and Glutamate Transmission in the Dorsal Striatum
Source: eNeuro. 2022 Apr 19;9(2):ENEURO.0119-22.2022. doi: 10.1523/ENEURO.0119-22.2022 (PMC9034757; doi:10.1523/ENEURO.0119-22.2022)

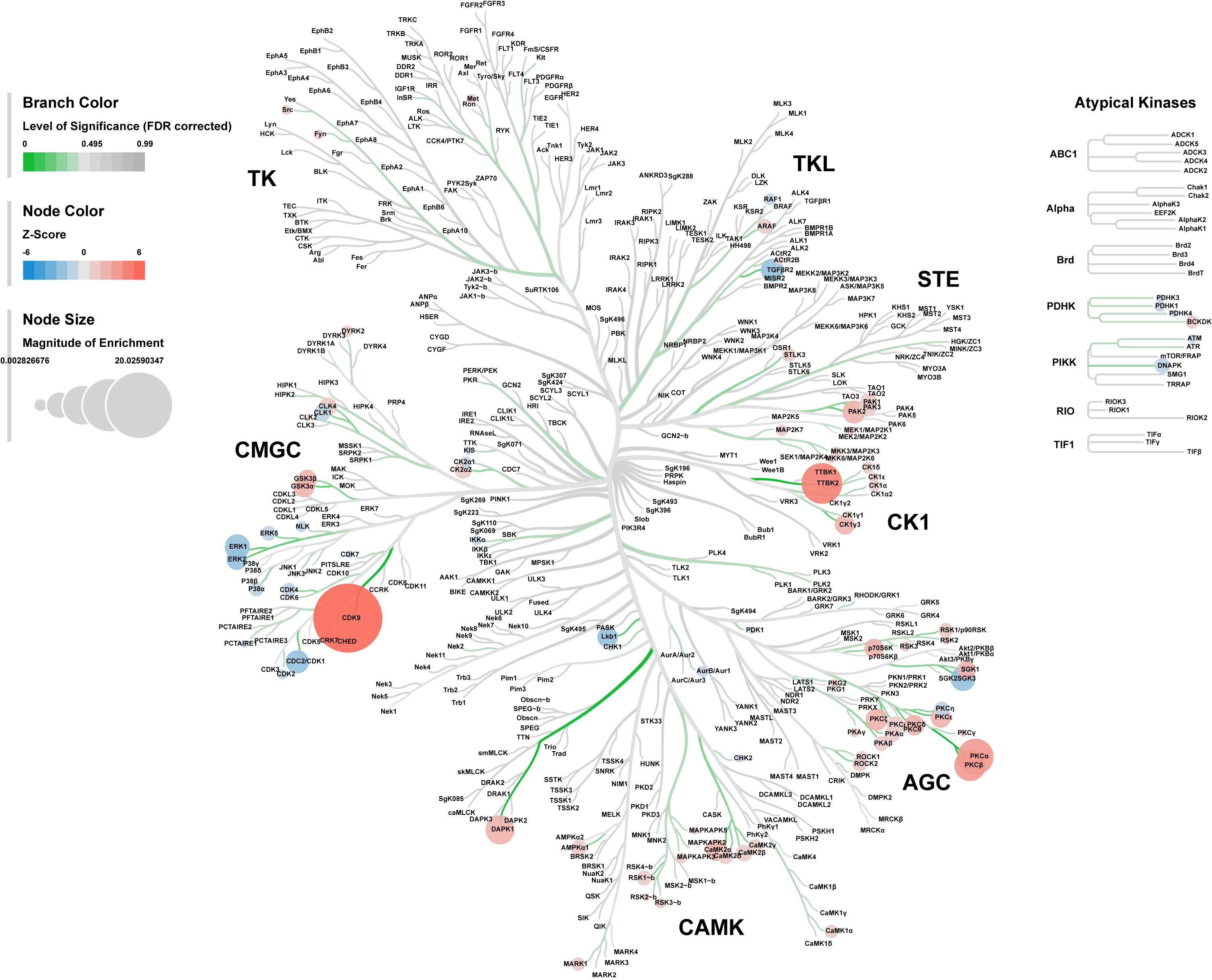

Supplement: Figure 2-4 — Kinome treeplots representing the full KSEA results for males in the DLS. The results from kinase-substrate enrichment analysis were mapped onto kinome treeplots via Coral in which branch color corresponds to significance level, node color corresponds to the z score of enrichment, and node size corresponds to the magnitude of enrichment for kinase pathways in the DLS of males. Download Figure 2-4, TIF file. [file enu-eN-NWR-0119-22-s08.tif]

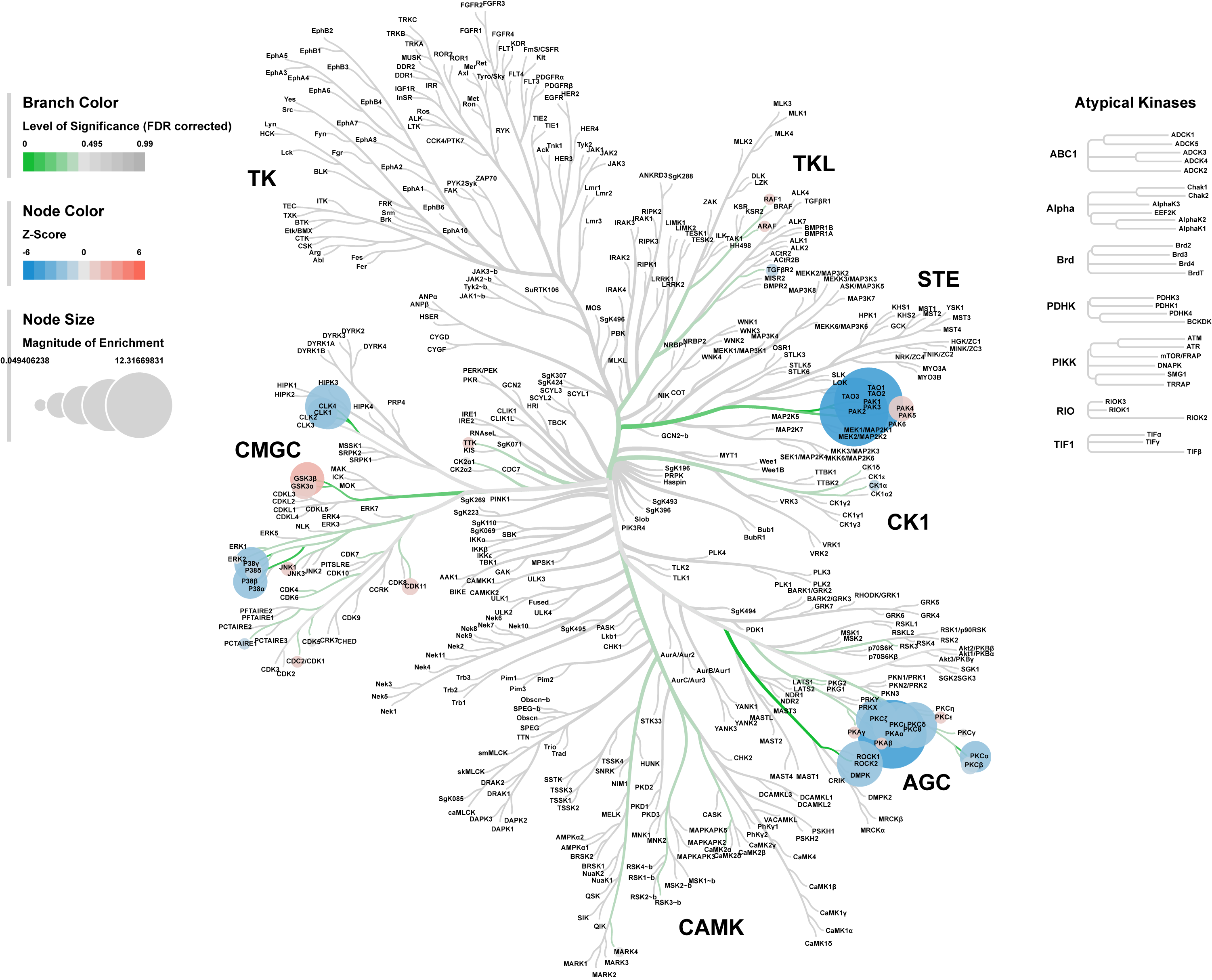

Supplement: Figure 2-5 — Kinome treeplots representing the full KSEA results for females in the DLS. The results from kinase-substrate enrichment analysis were mapped onto kinome treeplots via Coral in which branch color corresponds to significance level, node color corresponds to the z score of enrichment, and node size corresponds to the magnitude of enrichment for kinase pathways in the DLS of females. Download Figure 2-5, TIF file. [file enu-eN-NWR-0119-22-s09.tif]

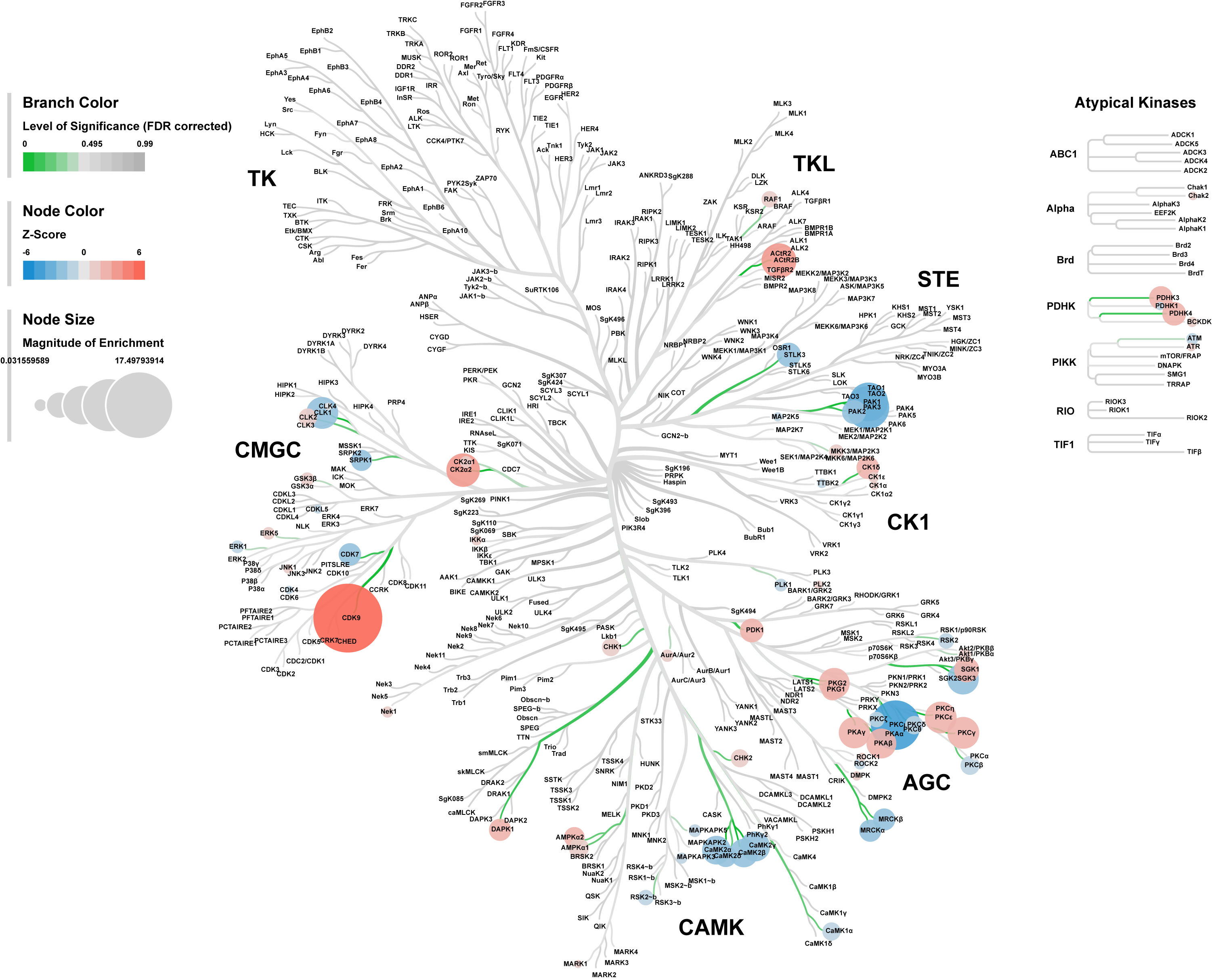

Supplement: Figure 2-6 — Kinome treeplots representing the full KSEA results for males in the DMS. The results from kinase-substrate enrichment analysis were mapped onto kinome treeplots via Coral in which branch color corresponds to significance level, node color corresponds to the z score of enrichment, and node size corresponds to the magnitude of enrichment for kinase pathways in the DMS of males. Download Figure 2-6, TIF file. [file enu-eN-NWR-0119-22-s10.tif]

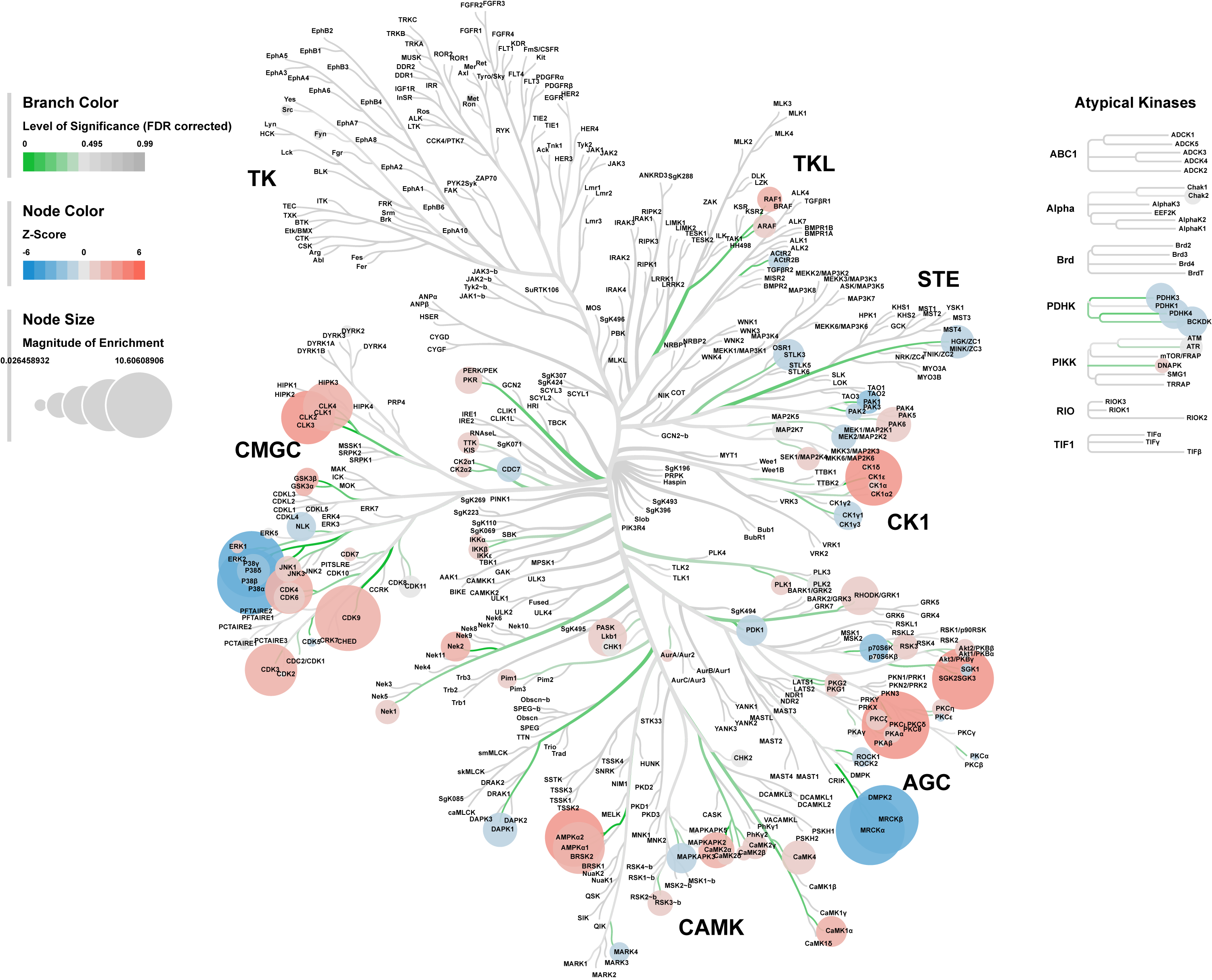

Supplement: Figure 2-7 — Kinome treeplots representing the full KSEA results for females in the DMS. The results from kinase-substrate enrichment analysis were mapped onto kinome treeplots via Coral in which branch color corresponds to significance level, node color corresponds to the z score of enrichment, and node size corresponds to the magnitude of enrichment for kinase pathways in the DMS of females. Download Figure 2-7, TIF file. [file enu-eN-NWR-0119-22-s11.tif]
